# Influencing factors for rapidly progressive interstitial lung disease in patients with anti-MDA5 antibody-positive dermatomyositis: a systematic review and meta-analysis

**DOI:** 10.3389/fimmu.2026.1796496

**Published:** 2026-04-10

**Authors:** Haixia Li, Mingyang Yi, Gao Da, Minghao Li, Zhiwan Wang

**Affiliations:** 1 Department of Respiratory Medicine, The First Affiliated Hospital of Henan University of Chinese Medicine, Zhengzhou, Henan, China; 2 The First Clinical Medical College, Henan University of Chinese Medicine, Zhengzhou, Henan, China; 3 Collaborative Innovation Center for Chinese Medicine and Respiratory Diseases Co-constructed by Henan Province and Ministry of Education of P. R. China, Henan University of Chinese Medicine, Zhengzhou, Henan, China; 4 Department of Endocrinology Medicine, The Third Affiliated Hospital of Henan University of Chinese Medicine, Zhengzhou, Henan, China

**Keywords:** anti-MDA5 antibody-positive dermatomyositis, influencing factors, meta-analysis, rapidly progressive interstitial lung disease, systematic review

## Abstract

**Background:**

The development of rapidly progressive interstitial lung disease (RP-ILD) in patients with anti-MDA5-positive dermatomyositis-associated interstitial lung disease (MDA5+ DM-ILD) is a major cause of adverse outcomes, including mortality. This study aimed to identify factors influencing the occurrence of RP-ILD in patients with MDA5+ DM-ILD.

**Methods:**

A systematic search was conducted in PubMed, EMBASE, the Cochrane Library, Web of Science, and Scopus for studies investigating factors associated with RP-ILD in MDA5+ DM-ILD, with the search period extending to January 1, 2026. The quality of the included studies was assessed using the Newcastle-Ottawa Scale (NOS). Meta-analysis was performed using Stata 18.0 software.

**Results:**

Fifteen studies were included in the meta-analysis, all of which were of high quality, with an average NOS score of 7.9. The meta-analysis revealed that male sex (OR = 1.99, 95% CI: 1.27-3.12), advanced age (OR = 2.54, 95% CI: 1.40-4.60), disease duration <3 months (OR = 3.23, 95% CI: 2.23-4.70), fever (OR = 2.46, 95% CI: 1.55-3.90), anti-Ro52 antibody positivity (OR = 5.05, 95% CI: 3.21-7.96), elevated C-reactive protein (CRP) (OR = 2.29, 95% CI: 1.78-2.94), elevated neutrophil-to-lymphocyte ratio (NLR) (OR = 2.29, 95% CI: 1.28-4.08), elevated lactate dehydrogenase (LDH) (OR = 3.39, 95% CI: 2.20-5.22), elevated aspartate aminotransferase (AST) (OR = 1.03, 95% CI: 1.00-1.06), elevated alanine aminotransferase (ALT) (OR = 2.42, 95% CI: 1.22-4.80), elevated serum ferritin (SF) (OR = 3.81, 95% CI: 1.95-7.42), lymphopenia (OR = 2.14, 95% CI: 1.10-4.16), and elevated carcinoembryonic antigen (CEA) (OR = 3.38, 95% CI: 1.32-8.66) were risk factors for RP-ILD. Conversely, arthralgia/arthritis (OR = 0.26, 95% CI: 0.16-0.44) and lymphocytosis (OR = 0.17, 95% CI: 0.09-0.31) were identified as potential protective factors.

**Conclusion:**

This study explored potential risk and protective factors associated with the development of RP-ILD in patients with MDA5+ DM-ILD, providing a basis for early identification and management.

**Systematic review registration:**

https://www.crd.york.ac.uk/prospero/, identifier CRD420261287925.

## 1 Introduction

Idiopathic inflammatory myopathies represent a group of autoimmune diseases characterized by skeletal muscle inflammation. Among them, dermatomyositis (DM) demonstrates distinctive clinical phenotypes due to its characteristic skin lesions and muscle involvement (1). In recent years, with advancements in autoantibody detection techniques, myositis-specific antibodies have emerged as crucial biomarkers for DM subtype classification, clinical phenotype prediction, and prognosis assessment. The discovery of anti-melanoma differentiation-associated gene 5 (MDA5) antibody has provided vital evidence for precise diagnosis and treatment of DM (2). Anti-MDA5-positive patients often present with prominent mucocutaneous lesions, less prominent myopathy, and heightened susceptibility to pulmonary involvement. This condition is particularly prevalent among Asian populations, characterized by rapid disease progression and extremely poor prognosis (3). Interstitial lung disease constitutes one of the primary factors contributing to poor prognosis in anti-MDA5-positive dermatomyositis patients. It frequently manifests as rapid deterioration of respiratory function early in disease onset, leading to respiratory failure and elevated risk of mortality. Relevant studies indicate that 35%–55% of anti-MDA5-positive DM-ILD patients are susceptible to acute exacerbation of pulmonary symptoms, with approximately 25% of patients experiencing mortality (4–6).

Acute exacerbation (AE) is a critical form of interstitial lung disease (ILD) progression, characterized by rapid onset of dyspnea, accelerated deterioration on pulmonary imaging, and hypoxemia. Patients often die from acute respiratory failure, with a 6-month survival rate of only 40.8%~54.5% (7). Studies indicate that patients with anti-MDA5-positive dermatomyositis-associated ILD face significantly higher risks of acute exacerbation compared to other myositis-related ILD subtypes. The pathogenesis involves complex mechanisms potentially linked to aberrant interferon pathway activation, dysregulated immune cell responses, and cytokine storm phenomena (8). Recent single-cell sequencing studies have confirmed significant interferon signaling activation in anti-MDA5-positive dermatomyositis patients with interstitial lung disease. Excessive interferon induces natural killer (NK) cell apoptosis and depletion, thereby promoting the progression of pulmonary inflammation (9). It should be noted that rapidly progressive interstitial lung disease (RP-ILD) is the leading cause of short-term mortality in anti-MDA5-positive DM-ILD patients. RP-ILD is typically defined as a rapid worsening of dyspnea within a short period (usually within 3 months of disease onset), accompanied by accelerated progression of interstitial lesions on high-resolution computed tomography. When necessary, other causes of acute respiratory deterioration such as infection, heart failure, pulmonary embolism, and drug-induced lung injury must be excluded (10). The pathogenesis of RP-ILD has not been fully elucidated and is currently thought to be associated with multiple factors including aberrant autoimmune responses, cytokine storms, rapid progression of pulmonary fibrosis, and infection (11). When RP-ILD occurs in anti-MDA5-positive DM patients with interstitial lung disease, they often present with rapidly worsening respiratory symptoms. Some patients may experience systemic manifestations such as fever and worsening cutaneous rash. These patients typically respond poorly to conventional glucocorticoids combined with immunosuppressants, with limited clinical treatment options. Within 6 months after RP-ILD onset, 50%-70% of patients die, demonstrating significantly higher mortality compared to non-rapidly progressive ILD patients (4, 12). Furthermore, the clinical identification of RP-ILD poses challenges, as some patients exhibit atypical imaging findings in the early stages, which can be easily confused with conditions such as pulmonary infections and acute respiratory distress syndrome, potentially delaying optimal treatment timing. In recent years, with the advancement of clinical research, researchers have increasingly focused on identifying factors influencing the development of RP-ILD in anti-MDA5-positive DM patients with interstitial lung disease, aiming to achieve early warning and intervention through the recognition of high-risk factors to improve patient prognosis. Reported potential influencing factors currently include demographic characteristics, clinical manifestations, laboratory parameters, imaging findings, baseline pulmonary function levels, and treatment regimens (13–15). However, existing studies are predominantly single-center, small-sample retrospective analyses with limited sample sizes and significant heterogeneity in study populations. Conclusions regarding influencing factors across different studies show inconsistencies, and results for certain factors remain controversial, lacking validation through large-sample, high-quality evidence-based medical evidence.

This study integrates existing research findings through systematic review and meta-analysis, utilizing quantitative synthesis to minimize bias inherent in individual studies, thereby enhancing the reliability and applicability of conclusions. Therefore, this study systematically synthesizes and quantitatively analyzes potential influencing factors to clarify the association strength between each factor and RP-ILD occurrence. This provides evidence-based medical support for early clinical identification of high-risk patients and the development of individualized preventive and therapeutic strategies, while also offering insights for future research directions.

## 2 Methods

This study strictly adhered to the Meta-analysis of Observational Studies in Epidemiology (MOOSE) statement (16) and the Preferred Reporting Items for Systematic Reviews and Meta-Analyses (PRISMA) guidelines (17). The study protocol was preregistered with the International Prospective Register of Systematic Reviews (PROSPERO) (CRD420261287925).

### 2.1 Search strategy

We systematically searched relevant articles in PubMed, EMBASE, Cochrane Library, Web of Science, and Scopus databases from inception to January 1, 2026, with no language restrictions. Two researchers independently performed the search. After deduplication, potentially relevant articles were screened based on titles and abstracts, followed by full-text evaluation. References of eligible articles were tracked to ensure comprehensive literature inclusion. Two independent reviewers jointly assessed the eligibility of the included studies through discussion. Any disagreements were resolved through consultation, with arbitration by a third researcher when necessary.

### 2.2 Inclusion/exclusion criteria

This review included English-language studies meeting the following criteria: (1) Population (P): Adult patients with anti-MDA5 antibody-positive DM-ILD; (2) Exposure (I): Clinical, demographic, imaging, physiological, serological, or treatment-related factors potentially associated with the development of RP-ILD in anti-MDA5 antibody-positive DM-ILD patients; (3) Comparison (C): Anti-MDA5 antibody-positive DM-ILD patients without the target exposure or who did not develop RP-ILD during follow-up; (4) Outcome (O): Factors associated with the development of RP-ILD in anti-MDA5 antibody-positive DM-ILD patients, reported as odds ratios (OR), risk ratios (RR), or hazard ratios (HR) with corresponding 95% confidence intervals (CI); (5) Study Design (S): Inclusion of cohort studies evaluating RP-ILD development in anti-MDA5 antibody-positive DM-ILD patients. Notably, mortality was not assessed as an outcome in this study. Exclusion criteria were as follows: (1) Duplicate publications (the version with the most complete data was prioritized for retention); (2) Literature with incomplete data or inability to extract target outcomes and corresponding data; (3) Reviews, letters, conference abstracts, case reports, editorials, and non-human studies; (4) Studies unpublished in peer-reviewed journals, such as dissertations, theses, and conference abstracts.

### 2.3 Data extraction and quality assessment

Using a predesigned standardized form, data were independently extracted by two researchers and cross-checked to ensure accuracy and completeness. Extracted information included Author, year, Study design, Country, Observation period, Age, Sample size, Outcome, Criteria for progression of interstitial lung disease and Incidence of worsening, Influencing factors. Regarding data on influencing factors, adjusted odds ratios (ORs) with 95% confidence intervals (CIs) for confounding factors were prioritized for extraction. For laboratory parameters, age, and other variables, we directly extracted the effect estimates reported in the original studies and adopted their definitions and analytical methods.

The quality of cohort studies was assessed using the Newcastle–Ottawa Scale (NOS) (18), covering three dimensions: subject selection, inter-group comparability, and outcome/exposure assessment, with a total score of 9 points. Two researchers independently scored the items, and any disagreements were discussed collectively and resolved by the third and fourth reviewers.

### 2.4 Data synthesis and statistical analysis

Meta-analysis was performed using Stata 18.0 statistical software. Effect sizes were expressed as odds ratios (OR) with 95% confidence intervals (CI). Heterogeneity among included studies was assessed using the χ2 test at a significance level of α=0.1, supplemented by I² statistics to quantitatively evaluate heterogeneity magnitude, with higher I² values indicating greater heterogeneity. If there was no statistical heterogeneity among the study results (P>0.1, I²<50%), a fixed-effects model was used for the meta-analysis; If statistical heterogeneity existed among the study results (P ≤ 0.1, I²≥50%), a random-effects model was used for the meta-analysis. For factors with heterogeneity, subgroup analysis was further conducted to explore the sources of heterogeneity and identify potential influencing factors. Sensitivity analysis and publication bias assessment were performed for factors with ≥3 included studies. Sensitivity analysis employed the leave-one-out method; if the pooled effect size and statistical significance remained substantially unchanged after removing any single study, it indicated that the meta-analysis results were stable; Publication bias was comprehensively evaluated using funnel plots, the trim-and-fill method, and Egger’s test. The overall significance level for the meta-analysis was set at α=0.05. For influencing factors mentioned in individual included studies that could not undergo quantitative analysis, they were summarized using descriptive analysis.

## 3 Results

### 3.1 Literature screening process and results

**Figure 1:**
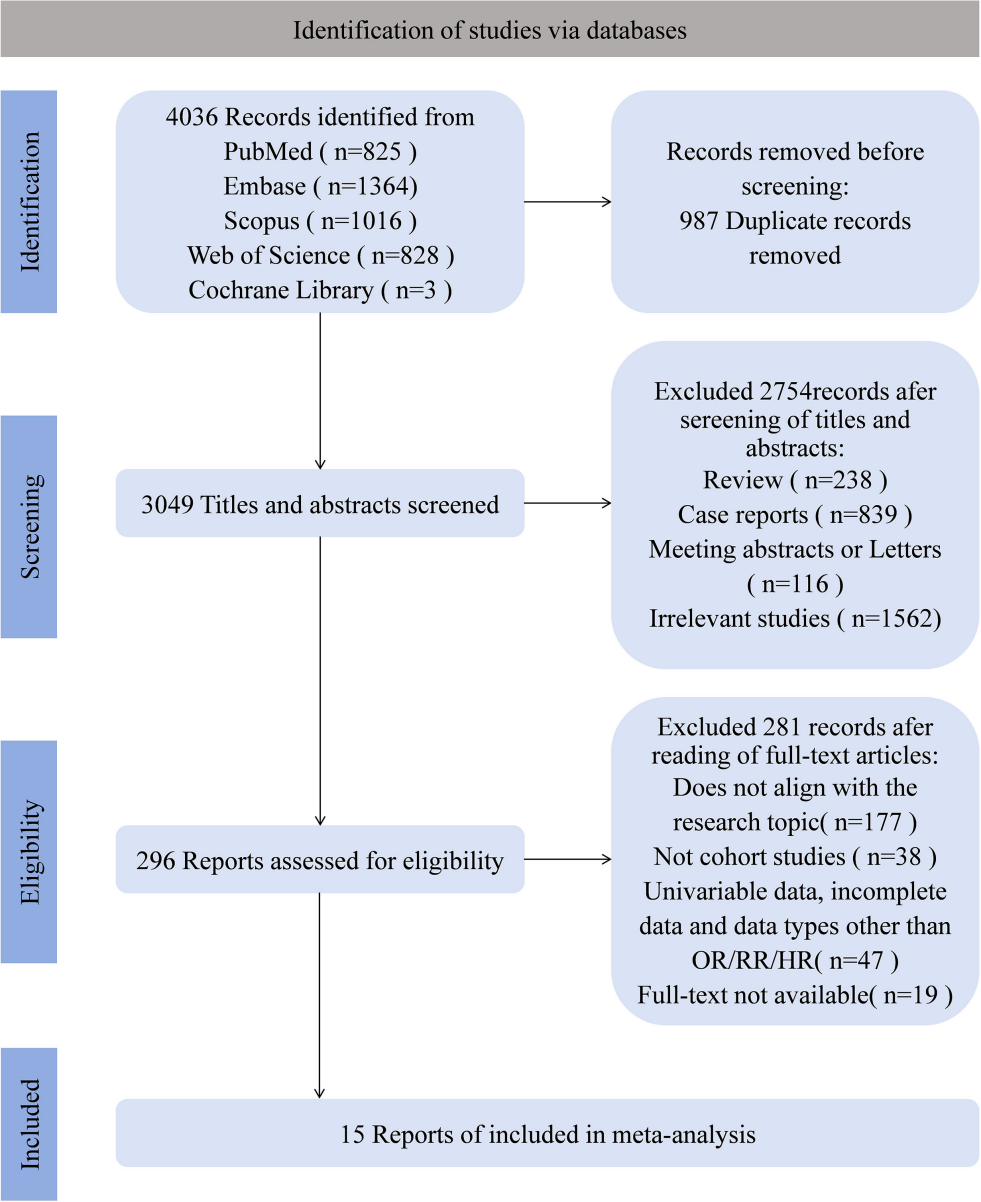
Flow diagram showing literature search and results.

A total of 4036 articles were retrieved, and after stepwise screening, 15 articles were finally included (4, 13, 19–31). The specific literature screening process is shown in **Figure 1**; **Supplementary Table 1**.

### 3.2 Basic characteristics and quality assessment of included studies

All 15 included articles were cohort studies, with a total sample size of 2233 cases. Thirty-two influencing factors identified through multivariate regression analysis were extracted, of which 16 were included in the meta-analysis and 16 underwent descriptive analysis. Detailed information is provided in **Supplementary Table 2**. The methodological quality of the included studies was evaluated using the NOS scale. The results showed that all 15 studies were of high quality (5 studies scored 7 points, 7 studies scored 8 points, and 3 studies scored 9 points), with an average NOS score of 7.9. Detailed scoring information is provided in **Supplementary Table 3**.

### 3.3 Analysis of related factors

#### 3.3.1 Demographic characteristics

##### 3.3.1.1 Male sex

A total of 5 studies were included (4, 19, 25, 27, 31). To assess the impact of male sex on the development of RP-ILD in MDA5^+^ DM patients, a fixed-effects model was used for meta-analysis to pool effect sizes since no significant heterogeneity was observed among the studies (p=0.357, I²=8.8%). Results indicated that male sex is a significant risk factor for RP-ILD development in MDA5^+^ DM patients (OR = 1.99, 95% CI: 1.27-3.12, p=0.003). Refer to **Table 1**; **Supplementary Figure 1**.

**Table 1:** Meta-analysis results.

Influencing factors	Included studies	Heterogeneity test		Effect model	Meta-analysis			Influence on RP-ILD
		I² (%)	p		OR	95% CI	p	
Demographic characteristics								
Male	5	8.8	0.357	Fixed	1.99	1.27-3.12	0.003	RF
Advanced age	5	74.5	0.003	Random	1.09	0.87-1.36	0.455	NS
Advanced age (dichotomous variable)	3	0	0.468	Fixed	2.54	1.40-4.60	0.002	RF
Advanced age (continuous variable)	2	76.9	0.038	Random	0.97	0.84-1.11	0.666	NS
Clinical characteristics								
Disease duration<3 months	3	0	0.617	Fixed	3.23	2.23-4.70	<0.001	RF
Fever	4	0	0.55	Fixed	2.46	1.55-3.90	<0.001	RF
Arthralgia/arthritis	3	38.4	0.197	Fixed	0.26	0.16-0.44	<0.001	PF
Rash	3	46.3	0.155	Fixed	0.72	0.39-1.34	0.301	NS
Immunological marker								
Anti-Ro52 positive	3	0	0.522	Fixed	5.05	3.21-7.96	<0.001	RF
Laboratory data								
Elevated CRP	8	0	0.690	Fixed	2.29	1.78-2.94	<0.001	RF
Elevated NLR	3	12.4	0.319	Fixed	2.29	1.28-4.08	0.005	RF
Elevated LDH	5	0	0.797	Fixed	3.39	2.20-5.22	<0.001	RF
Elevated AST	2	0	0.881	Fixed	1.03	1.00-1.06	0.020	RF
Elevated ALT	2	0	0.798	Fixed	2.42	1.22-4.80	0.012	RF
Elevated SF	2	0	0.415	Fixed	3.81	1.95-7.42	<0.001	RF
Lymphocytosis	2	0	0.690	Fixed	0.17	0.09-0.31	<0.001	PF
Lymphopenia	2	0	0.996	Fixed	2.14	1.10-4.16	0.025	RF
Elevated CEA	2	53.7	0.142	Random	3.38	1.32-8.66	0.011	RF

##### 3.3.1.2 Advanced age

A total of 5 studies were included (19, 20, 24, 27, 31). When evaluating the impact of advanced age on RP-ILD occurrence in MDA5^+^ DM patients, significant heterogeneity was observed across studies (p=0.003, I²=74.5%). A random-effects model was applied to pool effect sizes for meta-analysis. Results demonstrated no significant association between advanced age and RP-ILD development in MDA5^+^ DM patients (OR = 1.09, 95% CI: 0.87-1.36, p=0.455). Further subgroup analysis by variable type showed no significant heterogeneity in the dichotomous variable group (p=0.468, I²=0), with statistically significant meta-analysis results (OR = 2.54, 95% CI: 1.40-4.60, p=0.002). In contrast, the continuous variables group maintained substantial heterogeneity (p=0.038, I²=76.9%), showing non-significant results (OR = 0.97, 95% CI: 0.84-1.11, p=0.666). This suggests that when analyzed as a dichotomous variable, advanced age demonstrates relatively more stable predictive effect for RP-ILD in MDA5^+^ DM patients. Notably, the two studies in the continuous variables group exhibited opposite effect directions—one identifying advanced age as a risk factor while the other as a protective factor for RP-ILD development. This discrepancy may affect the stability of pooled results and constitute a potential source of heterogeneity. See **Table 1**, **Supplementary Figure 1**.

#### 3.3.2 Clinical features

##### 3.3.2.1 Disease duration <3 months

Three studies were included (4, 23, 26). To evaluate the impact of disease duration <3 months on RP-ILD development in MDA5^+^ DM patients, meta-analysis was performed using the fixed-effects model to pool effect sizes since no significant heterogeneity existed among studies (p=0.617, I²=0). The results demonstrated that disease duration <3 months was a risk factor for RP-ILD in MDA5^+^ DM patients (OR = 3.23, 95% CI: 2.23-4.70, p<0.001). Refer to **Table 1**; **Supplementary Figure 2**.

##### 3.3.2.2 Fever

A total of 4 studies were included (20, 24, 29, 31). To evaluate the impact of fever on RP-ILD occurrence in MDA5^+^ DM patients, no significant heterogeneity was observed among studies (p=0.550, I²=0). A fixed-effects model was employed to conduct meta-analysis by pooling effect sizes. Results demonstrated that fever constitutes a risk factor for RP-ILD development in MDA5^+^ DM patients (OR = 2.46, 95% CI: 1.55-3.90, p<0.001). Refer to **Table 1**; **Supplementary Figure 2**.

##### 3.3.2.3 Arthralgia/arthritis

Three studies were included (24, 25, 29). To evaluate the impact of arthralgia/arthritis on RP-ILD occurrence in MDA5^+^ DM patients, no significant heterogeneity was observed among studies (p=0.197, I²=38.4%). A fixed-effects model was employed to conduct meta-analysis by pooling effect sizes. The results demonstrated that arthralgia/arthritis serves as a protective factor against RP-ILD in MDA5^+^ DM patients (OR = 0.26, 95% CI: 0.16-0.44, p<0.001). Refer to **Table 1**; **Supplementary Figure 2**.

##### 3.3.2.4 Cutaneous rash

Three studies were included (20, 24, 25). In assessing the impact of cutaneous rash on RP-ILD development in MDA5^+^ DM patients, no significant heterogeneity was observed across studies (p=0.155, I²=46.3%). A fixed-effects model was employed to pool effect sizes for meta-analysis. The analysis revealed no significant association between cutaneous rash and RP-ILD occurrence in MDA5^+^ DM patients (OR = 0.72, 95% CI: 0.39-1.34, p=0.301). Refer to **Table 1**; **Supplementary Figure 2**.

#### 3.3.3 Immunological markers

##### 3.3.3.1 Anti-Ro52 antibody positivity

Three studies were included (4, 25, 26). The impact of anti-Ro52 antibody positivity on the development of RP-ILD in MDA5^+^ DM patients was evaluated. No significant heterogeneity was observed among the studies (p=0.522, I²=0). A meta-analysis was conducted using the fixed-effects model to calculate the pooled effect size. The results demonstrated that anti-Ro52 antibody positivity is a risk factor for RP-ILD development in MDA5^+^ DM patients (OR = 5.05, 95% CI: 3.21-7.96, p<0.001). Refer to **Table 1**; **Supplementary Figure 3**.

#### 3.3.4 Laboratory parameters

##### 3.3.4.1 Elevated C-reactive protein

Eight studies were included (4, 19–21, 23, 26, 30, 31). To assess the impact of elevated CRP on RP-ILD development in MDA5^+^ DM patients, no significant heterogeneity was observed among studies (p=0.690, I²=0). A fixed-effects model was employed for meta-analysis to calculate the pooled effect size. Results indicated that elevated CRP was a risk factor for RP-ILD in MDA5^+^ DM patients (OR = 2.29, 95% CI: 1.78-2.94, p<0.001). See **Table 1**; **Supplementary Figure 4**.

##### 3.3.4.2 Elevated neutrophil-to-lymphocyte ratio

Three studies were included (19–21). To assess the impact of elevated NLR on RP-ILD development in MDA5^+^ DM patients, no significant heterogeneity was observed among studies (p=0.319, I²=12.4%). A fixed-effects model was employed for pooling effect sizes in the meta-analysis. Results demonstrated that elevated NLR served as a risk factor for RP-ILD in MDA5^+^ DM patients (OR = 2.29, 95% CI: 1.28-4.08, p=0.005). Refer to **Table 1**; **Supplementary Figure 4**.

##### 3.3.4.3 Elevated lactate dehydrogenase

A total of 5 studies were included (13, 20, 24, 29, 30). When evaluating the effect of elevated LDH on RP-ILD occurrence in MDA5^+^ DM patients, no significant inter-study heterogeneity was detected (p=0.797, I²=0). Effect sizes were pooled using a fixed-effects model for meta-analysis. Results indicated that elevated LDH is a risk factor for RP-ILD development in MDA5^+^ DM patients (OR = 3.39, 95% CI: 2.20-5.22, p<0.001). Refer to **Table 1**; **Supplementary Figure 4**.

##### 3.3.4.4 Elevated aspartate aminotransferase

Two studies were included (13, 27). When evaluating the impact of elevated AST on RP-ILD development in MDA5^+^ DM patients, no significant heterogeneity was observed among studies (p=0.881, I²=0). A fixed-effects model was employed to calculate the pooled effect size in the meta-analysis. Results demonstrated that elevated AST constitutes a risk factor for RP-ILD in MDA5^+^ DM patients (OR = 1.03, 95% CI: 1.00-1.06, p=0.020). Refer to **Table 1**; **Supplementary Figure 4**.

##### 3.3.4.5 Elevated alanine aminotransferase

Two studies were included (13, 29). The evaluation of elevated AST’s impact on RP-ILD development in MDA5^+^ DM patients revealed no significant heterogeneity among studies (p=0.798, I²=0). A meta-analysis was conducted using the fixed-effects model to pool effect sizes. Results indicated that elevated ALT constitutes a risk factor for RP-ILD in MDA5^+^ DM patients (OR = 2.42, 95% CI: 1.22-4.80, p=0.012). Refer to **Table 1**; **Supplementary Figure 4**.

##### 3.3.4.6 Serum ferritin

Two studies were included (28, 29). The assessment of elevated SF’s influence on RP-ILD occurrence in MDA5^+^ DM patients showed no significant inter-study heterogeneity (p=0.415, I²=0). Meta-analysis was performed using the fixed-effects model to pool effect sizes. The results demonstrated that elevated serum ferritin (SF) is a risk factor for RP-ILD development in MDA5^+^ DM patients (OR = 3.81, 95% CI: 1.95-7.42, p<0.001). See **Table 1**; **Supplementary Figure 4**.

##### 3.3.4.7 Lymphocytosis

Two studies were included (13, 22). When evaluating the impact of lymphocytosis on RP-ILD occurrence in MDA5^+^ DM patients, no significant heterogeneity was observed across studies (p=0.690, I²=0). A fixed-effects model was employed to calculate the pooled effect size for meta-analysis. The results indicated that lymphocytosis serves as a protective factor for RP-ILD development in MDA5^+^ DM patients (OR = 0.17, 95% CI: 0.09-0.31, p<0.001). See **Table 1**; **Supplementary Figure 4**.

##### 3.3.4.8 Lymphopenia

Two studies were included (24, 29). To evaluate the impact of lymphopenia on the development of RP-ILD in MDA5^+^ DM patients, meta-analysis was performed using a fixed-effects model to pool effect sizes, as no significant heterogeneity was observed among studies (p=0.996, I²=0). Results indicated that lymphopenia serves as a risk factor for RP-ILD in MDA5^+^ DM patients (OR = 2.14, 95% CI: 1.10-4.16, p=0.025). See **Table 1**; **Supplementary Figure 4**.

##### 3.3.4.9 Elevated carcinoembryonic antigen

Two studies were included (29, 30). To assess the effect of elevated CEA on RP-ILD development in MDA5^+^ DM patients, meta-analysis was conducted using a random-effects model to pool effect sizes, given the presence of heterogeneity among studies (p=0.142, I²=53.7%). The results indicated that elevated CEA is a risk factor for RP-ILD in MDA5^+^ DM patients (OR = 3.38, 95% CI: 1.32-8.66, p=0.011). Due to the inclusion of only two studies, subgroup analysis was not performed. The heterogeneity may be attributed to characteristics of the study population, CEA assay methods, and definitions of elevated thresholds, which require further investigation with reference to the original articles. See **Table 1**; **Supplementary Figure 4**.

### 3.4 Sensitivity analysis

Sensitivity analysis was conducted through sequential removal of individual studies for factors included in ≥3 studies. The results demonstrated that the risk factor of advanced age showed sensitivity: the OR fluctuated between 0.99-1.74 with 95% CI retaining statistical significance after removal of any single study; Grouped by variable type (continuous/dichotomous) with increasing age, the meta-analysis results remained stable in the dichotomous variable group. In the continuous variable group, only two studies lacked sensitivity analysis, suggesting that sensitivity originated from combined analyses of different definitions of age progression.

After sequentially excluding individual studies for other influencing factors, the direction and statistical significance of the pooled effect size showed no substantial changes, indicating stable meta-analysis results. It should be noted that the number of studies included for each influencing factor in this research was fewer than 10. Conclusions from sensitivity analyses should be interpreted with caution. See **Table 2**; **Supplementary Figures 5-8**.

**Table 2:** Sensitivity analysis and publication bias.

Influencing factors	Included studies	Sensitivity analysis	Effect model	Egger’s test		Trim-and-fill results		Bias
				t	p	OR	95% CI	
Demographic characteristics								
Male	5	Robust	Fixed	-0.54	0.628	1.99	1.27-3.12	No
Advanced age	5	Not robust	Random	1.67	0.193	0.98	0.75-1.27	No
Advanced age (dichotomous variable)	3	Robust	Fixed	-0.19	0.880	2.54	1.40-4.60	No
Advanced age (continuous variable)	2	NA	NA	NA	NA	NA	NA	NA
Clinical characteristics								
Disease duration<3 months	3	Robust	Fixed	5.83	0.110	2.78	2.04-3.81	No
Fever	4	Robust	Fixed	-0.63	0.595	2.46	1.55-3.90	No
Arthralgia/arthritis	3	Robust	Fixed	-2.03	0.291	0.26	0.16-0.44	No
Rash	3	Robust	Fixed	-0.33	0.795	0.72	0.39-1.34	No
Immunological marker								
Anti-Ro52 positive	3	Robust	Fixed	2.24	0.268	5.05	3.21-7.96	No
Laboratory data								
Elevated CRP	8	Robust	Fixed	-0.16	0.879	2.29	1.78-2.94	No
Elevated NLR	3	Robust	Fixed	1.48	0.378	2.29	1.28-4.08	No
Elevated LDH	5	Robust	Fixed	0.39	0.723	3.39	2.20-5.22	No
Elevated AST	2	NA	NA	NA	NA	NA	NA	NA
Elevated ALT	2	NA	NA	NA	NA	NA	NA	NA
Elevated SF	2	NA	NA	NA	NA	NA	NA	NA
Lymphocytosis	2	NA	NA	NA	NA	NA	NA	NA
Lymphopenia	2	NA	NA	NA	NA	NA	NA	NA
Elevated CEA	2	NA	NA	NA	NA	NA	NA	NA

Sensitivity analysis and bias testing were not performed for predictive factors included in only 2 studies (NA: not applicable).

### 3.5 Publication bias analysis

Publication bias assessment was performed for factors with ≥3 included studies. The p-values of Egger’s test were all < 0.05. The OR values adjusted by the trim-and-fill method showed little difference from the pooled OR values from the meta-analysis. The funnel plots were generally symmetric, with no studies requiring imputation, and the effect sizes did not change substantially after trim-and-fill adjustment. It is important to note that the number of studies included for each factor was less than 10. Therefore, the conclusions regarding publication bias should be interpreted with caution. Refer to **Table 2**; **Supplementary Figures 9-12**.

### 3.6 Factors not included in meta-analysis

For potential factors reported in only one study, descriptive analysis was employed due to the inability to perform pooling through meta-analysis. A total of 16 influencing factors were identified, including 13 risk factors and 3 protective factors. According to the type of indicator, they were classified into two categories: 15 were baseline-related indicators (including 12 risk factors and 3 factors associated with a lower likelihood of RP-ILD), and 1 was a treatment-related indicator (daily prednisone dose ≥67.5 mg). See **Table 3**.

**Table 3:** Summary of influencing factors not included in the meta-analysis.

Influencing factors	OR (95% CI)	p-value	Influence on RP-ILD
BAFF>2971.5 (pg/ml) (21)	9.39 (1.61-54.77)	0.013	RF
Elevated IL-6 (pg/mL) (22)	1.00 (1.00-1.00)	0.027	RF
Elevated WBC(×10^9^/L) (13)	1.54 (1.12-2.13)	0.009	RF
Elevated Oxygenation index (24)	0.98 (0.97-0.99)	<0.001	PF
Elevated S100A6 (100pg/mL) (27)	0.61 (0.43-0.86)	0.005	PF
WDFY4 rs7919656 variant (28)	4.98 (1.59-17.19)	0.008	RF
FVC% predicted<50% (28)	9.63 (4.12-25.16)	<0.001	RF
DLCO% predicted<30% (28)	2.76 (1.45-5.45)	0.002	RF
Prednisolone≥67.5 (mg/d) (28)	29.82 (9.04-124.70)	<0.001	RF
Decreased CD3+ T (29)	2.56 (1.17-5.61)	0.019	RF
Decreased CD3+CD4+ T (29)	2.80 (1.37-5.73)	0.005	RF
Decreased CD3+CD8+ T (29)	2.18 (1.05-4.50)	0.036	RF
Elevated B2 (29)	3.17 (1.41-7.13)	0.005	RF
Elevated CA153 (29)	3.31 (1.50-7.27)	0.003	RF
Decreased Nor LLZ (31)	0.99 (0.95-1.00)	0.009	PF
Increased Con LMZ (31)	1.02 (1.00-1.24)	0.002	RF

## 4 Discussion

Through systematic review and meta-analysis of 15 high-quality cohort studies involving 2,233 patients, this study comprehensively explored potential influencing factors for RP-ILD development in anti-MDA5-positive DM patients. Results identified 12 risk factors and 2 protective factors through meta-analysis of 16 included studies. Descriptive analysis supplemented findings by reporting 16 additional potential factors from single studies 13 risk factors and 3 protective factors). This provides an important evidence-based medical foundation for early clinical identification of high-risk patients and the formulation of personalized intervention strategies.

### 4.1 Factors related to demographic characteristics

The study found that male sex is an independent risk factor for RP-ILD in anti-MDA5-positive DM patients, with low heterogeneity across studies and stable sensitivity analysis results, suggesting that sex differences may play a significant role in RP-ILD. This finding aligns with the conclusion of the CROSS prediction model constructed by Wang L et al (4) which similarly lists male sex as one of the core risk factors for RP-ILD development. Physiological differences related to sex may influence immune response patterns; for instance, androgen levels in males may regulate the activation intensity of the interferon pathway (32), while the abnormal activation of interferon signaling pathways has been confirmed to be closely associated with the progression of pulmonary lesions in anti-MDA5-positive DM patients (33). Additionally, male patients may exhibit deficiencies in early symptom recognition and timeliness of medical consultation, indirectly contributing to rapid disease progression (34), a hypothesis that requires validation through prospective studies. Analysis of age factors revealed no significant correlation between advancing age and RP-ILD occurrence overall. However, subgroup analysis indicated that when age was treated as a dichotomous variable (using a fixed age cutoff), its predictive effect reached statistical significance. In contrast, results from the grouped continuous variables showed no significance and demonstrated high heterogeneity. This discrepancy may stem from varying definitions of “advanced age” across studies, leading to inconsistencies in pooled results. Notably, in the grouped continuous variables analysis, Yan W et al (27) the study indicated that patients with younger age of onset were more prone to developing RP-ILD, suggesting that advanced age serves as a protective factor against RP-ILD occurrence. This paradoxical finding contradicts established clinical consensus and other research conclusions, representing the primary source of heterogeneity in this subgroup. It is hypothesized that this protective effect may arise from age-related physiological decline in immune function, which attenuates the intensity of anti-MDA5 antibody-mediated hyperinflammatory responses, thereby reducing the risk of acute lung tissue injury (35). Moreover, elderly patients often receive more attention regarding their clinical manifestations, potentially enabling earlier intervention therapies that may delay the progression of RP-ILD. However, this conclusion has certain limitations. Future studies need to establish a consensus on the definition and analytical approach for the age variable while expanding sample sizes to validate its actual relationship with RP-ILD.

### 4.2 Factors related to clinical features

The study found that a disease course <3 months is a significant risk factor for RP-ILD development, which highly consistent with the definition of RP-ILD (rapid progression within 3 months of onset). This validates the clinical characteristics of acute onset and rapid progression in anti-MDA5-positive DM-ILD (35). Insufficient early intervention may represent a critical reason for rapid progression in patients with short disease duration (4). Therefore, for anti-MDA5-positive DM patients, even in the absence of obvious pulmonary symptoms, close monitoring should be initiated immediately after diagnosis to prevent treatment delay. Disease duration <3 months was associated with a higher risk of RP-ILD. Although potential circular definition may exist due to overlapping time windows, the reference point was clearly defined as the onset time of isolated ILD in this study, and the results were stable as verified by sensitivity analysis. Therefore, this association still has clinical reference value, and comprehensive evaluation combined with other indicators is recommended in clinical practice. Fever serves as another significant clinical risk factor, potentially linked to systemic cytokine storm mechanisms. Prior research confirms that during fever episodes in anti-MDA5-positive DM patients, serum levels of pro-inflammatory cytokines such as IL-6 and TNF-α significantly elevate and demonstrate positive correlation with disease activity in pulmonary lesions (2). Notably, arthralgia/arthritis has been identified as a protective factor against RP-ILD development. This phenomenon may be attributed to the observed inverse correlation between joint involvement and muscle involvement in anti-MDA5-positive DM patients. Patients with prominent myopathic manifestations demonstrate a relatively lower risk of pulmonary involvement. It is hypothesized that the presence of arthralgia/arthritis may reflect a ‘targeting difference’ in immune responses, whereby immune complexes preferentially deposit in joint tissues, thereby mitigating damage to pulmonary parenchyma (36). Furthermore, articular symptoms may prompt earlier medical consultation, indirectly facilitating timely intervention and reducing RP-ILD risk. Our study found no significant correlation between cutaneous rash and RP-ILD, indicating that rash cannot serve as a predictor for pulmonary lesion progression. This finding diverges from certain studies suggesting ‘patients with severe rashes have poorer prognoses’ (25), potentially attributable to variations in rash phenotypes (such as differing pathological implications of Gottron’s sign, heliotrope rash, etc.), warranting further analysis for verification.

### 4.3 Factors related to immunological markers

Anti-Ro52 antibody positivity emerged as a high-risk factor in this study, suggesting that co-positivity of anti-Ro52 and anti-MDA5 antibodies may significantly exacerbate disease severity, consistent with findings by Cheng L et al (25). This aligns with pathological research findings which demonstrate that in lung tissues of patients dually positive for anti-Ro52 and anti-MDA5 antibodies, neutrophil infiltration and fibrosis severity are significantly higher than in single antibody-positive patients. Anti-Ro52 antibodies may amplify the inflammatory response by enhancing type I interferon pathway activation, forming a synergistic pathogenic effect with anti-MDA5 antibodies. Therefore, combined testing for anti-Ro52 antibodies can significantly improve the accuracy of RP-ILD risk prediction (37).

### 4.4 Laboratory parameter-related factors

Multiple laboratory parameters have been confirmed as risk factors for RP-ILD development, including elevated CRP, LDH, SF, NLR, AST, and ALT. These factors demonstrated low heterogeneity across studies and stable sensitivity analysis results, suggesting they may play significant roles in RP-ILD pathogenesis. As a classic acute-phase reactant, elevated CRP levels reflect the intensity of systemic inflammatory response and are closely associated with pulmonary inflammatory infiltration and disease progression (38); LDH is primarily found in lung parenchymal cells, and its elevation reflects the extent of pulmonary tissue damage and cellular necrosis (39); As an acute-phase protein, SF synthesis increases under cytokine stimulation and correlates with the progression of pulmonary fibrosis (40); NLR serves as a novel inflammatory marker that reflects increased neutrophil infiltration and lymphocyte depletion caused by immune-inflammatory dysregulation and oxidative stress stimulation, which is closely associated with disease severity and poor prognosis (41); Liver function markers (AST, ALT) suggest that anti-MDA5-positive DM patients may exhibit multi-organ involvement, where hepatic injury and pulmonary lesions potentially share common immunopathological mechanisms (42). These parameters are accessible and cost-effective for testing, making them suitable as routine clinical monitoring indicators for RP-ILD risk stratification. Analysis of lymphocyte-related parameters revealed lymphocytosis as a protective factor against RP-ILD development in anti-MDA5-positive DM patients, whereas lymphopenia constituted a risk factor. This finding aligns with the theory of immune cell dysregulation (43). Sufficient lymphocytes effectively maintain the body’s immune homeostasis, thereby preventing the occurrence and progression of abnormal inflammatory responses. Elevated carcinoembryonic antigen (CEA) as a risk factor may reflect the activation of abnormal pulmonary tissue repair and fibrotic processes. Existing studies have confirmed that serum CEA levels are positively correlated with the severity of pulmonary fibrosis in patients with interstitial lung disease (44). Combined research findings indicate that both included studies identified elevated CEA as a risk factor for RP-ILD, but with moderate heterogeneity. This may be attributed to differences in study populations and varying threshold definitions for CEA elevation. Subsequent multicenter studies should standardize testing criteria and definitions to further validate the predictive value of CEA. Of note, some laboratory markers (such as AST) show statistical significance but have OR values close to 1, indicating weak associations with limited clinical practical value. This suggests that these indicators should be comprehensively evaluated in combination with other clinical information when used for risk assessment, to avoid over−reliance on a single marker.

### 4.5 Potential factors not included in meta-analysis

The descriptive analysis incorporated 16 potential risk factors, among which FVC% <50% and a daily prednisone dose ≥67.5mg demonstrated particularly strong associations, with OR values reaching 9.63 and 29.82 respectively. This suggests that severe impairment of pulmonary function and high-dose glucocorticoid administration may serve as key influencing factors for RP-ILD development in anti-MDA5-positive DM patients. A significant decrease in FVC% reflects severe insufficiency in baseline pulmonary functional reserve and pre-existing irreversible damage to lung parenchymal structures. Such compromised lungs demonstrate diminished tolerance to subsequent acute inflammatory insults, consequently accelerating the progression of pulmonary lesions (45); Moreover, the long-term use of high-dose prednisone may indirectly promote the rapid progression of interstitial lung disease through pathways such as excessive suppression of immune surveillance, induction of immune response imbalance, or increased risk of superinfection. However, the use of high-dose steroids more likely reflects the severity of the patients’ baseline pulmonary condition, and a direct causal role of steroids in driving disease progression cannot be confirmed (46). Additionally, immune-related indicators such as elevated serum interleukin-6 (IL-6) levels and reduced peripheral blood CD3^+^ T-lymphocyte counts are also potential risk factors for RP-ILD occurrence. This finding further corroborates the central role of cytokine storms and imbalance in immune cell subsets in the pathogenesis of RP-ILD (47). It is noteworthy that the association effects of the aforementioned immune-related indicators have currently been reported in only a single study. There remains a lack of replicated validation through multicenter, large-sample cohort studies. Their clinical value and predictive efficacy still require further clarification in subsequent research.

### 4.6 Limitations of this study

(1) All included studies were cohort studies, and the study populations were all from China. Some studies had a single-center, small-sample design, which may lead to selection bias; therefore, caution should be exercised when generalizing the conclusions. (2) The number of included studies for each predictor was small (all < 10), resulting in limited stability and reliability of the meta-analysis results. The statistical power of sensitivity analysis and publication bias assessment was insufficient, and potential publication bias may exist. (3) Differences in the diagnostic criteria of RP-ILD, the definition of laboratory marker thresholds, and variable processing methods across studies may represent an important source of heterogeneity. (4) ILD imaging types (e.g., UIP pattern, NSIP pattern, mixed pattern, etc.) were not included in the analysis, since the included studies did not report consistent and sufficient data on imaging classification to enable a reliable meta-analysis. (5) Non-English articles were not included, which may lead to potential literature omission. Future studies should conduct multicenter, prospective cohort studies, unify diagnostic criteria and variable definitions, further validate the identified influencing factors, and explore novel predictive indicators, so as to lay a foundation for developing risk stratification tools and achieving more precise identification of high-risk patients.

In summary, the influencing factors for RP-ILD development in anti-MDA5-positive DM patients involve multiple dimensions including demographic characteristics, clinical manifestations, immunological markers, and laboratory parameters. These findings support clinicians in comprehensively evaluating patients, identifying high-risk populations for RP-ILD at an early stage, and implementing individualized interventions to reduce the incidence and mortality of RP-ILD. Further high-quality studies are needed to explore the pathogenic mechanisms of these factors and provide more robust evidence for optimizing clinical decision-making.

## Data Availability

The original contributions presented in the study are included in the article/**Supplementary Material**. Further inquiries can be directed to the corresponding author.
