# Supplementary material for: Influencing factors for rapidly progressive interstitial lung disease in patients with anti-MDA5 antibody-positive dermatomyositis: a systematic review and meta-analysis
Source: Front Immunol. 2026 Apr 10;17:1796496. doi: 10.3389/fimmu.2026.1796496 (PMC13106528; doi:10.3389/fimmu.2026.1796496)
Supplement: Supplementary file 1 [file Supplementaryfile1.docx]

Supplementary Material

**Table of Contents for Supplementary Material**

[1 Supplementary Table 1: Search Strategy 2](#_Toc220200763)

[2 Supplementary Table 2: Basic Characteristics of Included Studies 4](#_Toc220200764)

[3 Supplementary Table 3: Results of Newcastle-Ottawa quality assessment Scale for each included study 7](#_Toc220200765)

[4 Meta-Analysis Results 8](#_Toc220200766)

[4.1 Demographic characteristics 8](#_Toc220200767)

[4.2 Clinical characteristics 8](#_Toc220200768)

[4.3 Immunological marker 8](#_Toc220200769)

[4.4 Laboratory data 9](#_Toc220200770)

[5 Sensitivity analysis 11](#_Toc220200771)

[5.1 Demographic characteristics 11](#_Toc220200772)

[5.2 Clinical characteristics 11](#_Toc220200773)

[5.3 Immunological marker 12](#_Toc220200774)

[5.4 Laboratory data 12](#_Toc220200775)

[6 Bias Assessment 13](#_Toc220200776)

[6.1 Demographic characteristics 13](#_Toc220200777)

[6.2 Clinical characteristics 14](#_Toc220200778)

[6.3 Immunological marker 15](#_Toc220200779)

[6.4 Laboratory data 15](#_Toc220200780)

# Supplementary Table 1: Search Strategy

Search strategies for the different databases ran on **January 1, 2026**. This strategy is adapted to identify trials in other electronic databases

PubMed (825)

| Search number | Query | Search Details | Results |
| --- | --- | --- | --- |
|  | (((Dermatomyositis[MeSH Terms]) OR (Dermatomyositis [Title/Abstract] OR Polymyositis-Dermatomyositis [Title/Abstract] OR Polymyositis Dermatomyositis [Title/Abstract] OR Dermatopolymyositis [Title/Abstract] OR Dermatomyositis, Adult Type [Title/Abstract] OR Adult Type Dermatomyositis [Title/Abstract] OR Dermatomyositis, Childhood Type [Title/Abstract] OR Childhood Type Dermatomyositis [Title/Abstract] OR Juvenile Dermatomyositis [Title/Abstract] OR Dermatomyositis, Juvenile [Title/Abstract] OR Juvenile Myositis [Title/Abstract] OR Myositis, Juvenile [Title/Abstract])) AND ((Interstitial Lung Diseases[MeSH Terms]) OR (Diffuse Parenchymal Lung Diseases [Title/Abstract] OR Interstitial Lung Diseases [Title/Abstract] OR Interstitial Lung Disease [Title/Abstract] OR Lung Disease, Interstitial [Title/Abstract] OR Diffuse Parenchymal Lung Disease [Title/Abstract] OR Pneumonia, Interstitial [Title/Abstract] OR Interstitial Pneumonia [Title/Abstract] OR Interstitial Pneumonias [Title/Abstract] OR Pneumonias, Interstitial [Title/Abstract] OR Pneumonitis, Interstitial [Title/Abstract] OR Interstitial Pneumonitides [Title/Abstract] OR Interstitial Pneumonitis [Title/Abstract] OR Pneumonitides, Interstitial [Title/Abstract]))) AND (anti-MDA5 antibody [Title/Abstract] OR MDA5 [Title/Abstract] OR MDA-5 [Title/Abstract] OR melanoma differentiation-associated gene-5 [Title/Abstract] OR anti-MDA5 [Title/Abstract]) | ("Dermatomyositis"[MeSH Terms] OR ("Dermatomyositis"[Title/Abstract] OR "Polymyositis-Dermatomyositis"[Title/Abstract] OR "Polymyositis-Dermatomyositis"[Title/Abstract] OR "Dermatopolymyositis"[Title/Abstract] OR (("Dermatomyositis"[MeSH Terms] OR "Dermatomyositis"[All Fields]) AND "adult type"[Title/Abstract]) OR "adult type dermatomyositis"[Title/Abstract] OR "dermatomyositis childhood type"[Title/Abstract] OR "childhood type dermatomyositis"[Title/Abstract] OR "juvenile dermatomyositis"[Title/Abstract] OR "dermatomyositis juvenile"[Title/Abstract] OR "juvenile myositis"[Title/Abstract] OR "myositis juvenile"[Title/Abstract])) AND ("lung diseases, interstitial"[MeSH Terms] OR ("diffuse parenchymal lung diseases"[Title/Abstract] OR "interstitial lung diseases"[Title/Abstract] OR "interstitial lung disease"[Title/Abstract] OR "lung disease interstitial"[Title/Abstract] OR "diffuse parenchymal lung disease"[Title/Abstract] OR "pneumonia interstitial"[Title/Abstract] OR "interstitial pneumonia"[Title/Abstract] OR "interstitial pneumonias"[Title/Abstract] OR "pneumonias interstitial"[Title/Abstract] OR "pneumonitis interstitial"[Title/Abstract] OR "interstitial pneumonitides"[Title/Abstract] OR "interstitial pneumonitis"[Title/Abstract] OR (("Pneumonia"[MeSH Terms] OR "Pneumonia"[All Fields] OR "Pneumonitides"[All Fields]) AND "Interstitial"[Title/Abstract]))) AND ("anti mda5 antibody"[Title/Abstract] OR "MDA5"[Title/Abstract] OR "MDA-5"[Title/Abstract] OR "melanoma differentiation associated gene 5"[Title/Abstract] OR "anti-MDA5"[Title/Abstract]) | 825 |
| 7 | anti-MDA5 antibody [Title/Abstract] OR MDA5 [Title/Abstract] OR MDA-5 [Title/Abstract] OR melanoma differentiation-associated gene-5 [Title/Abstract] OR anti-MDA5 [Title/Abstract] | "anti mda5 antibody"[Title/Abstract] OR "MDA5"[Title/Abstract] OR "MDA-5"[Title/Abstract] OR "melanoma differentiation associated gene 5"[Title/Abstract] OR "anti-MDA5"[Title/Abstract] | 3,448 |
| 6 | (Interstitial Lung Diseases[MeSH Terms]) OR (Diffuse Parenchymal Lung Diseases [Title/Abstract] OR Interstitial Lung Diseases [Title/Abstract] OR Interstitial Lung Disease [Title/Abstract] OR Lung Disease, Interstitial [Title/Abstract] OR Diffuse Parenchymal Lung Disease [Title/Abstract] OR Pneumonia, Interstitial [Title/Abstract] OR Interstitial Pneumonia [Title/Abstract] OR Interstitial Pneumonias [Title/Abstract] OR Pneumonias, Interstitial [Title/Abstract] OR Pneumonitis, Interstitial [Title/Abstract] OR Interstitial Pneumonitides [Title/Abstract] OR Interstitial Pneumonitis [Title/Abstract] OR Pneumonitides, Interstitial [Title/Abstract]) | "lung diseases, interstitial"[MeSH Terms] OR ("diffuse parenchymal lung diseases"[Title/Abstract] OR "interstitial lung diseases"[Title/Abstract] OR "interstitial lung disease"[Title/Abstract] OR "lung disease interstitial"[Title/Abstract] OR "diffuse parenchymal lung disease"[Title/Abstract] OR "pneumonia interstitial"[Title/Abstract] OR "interstitial pneumonia"[Title/Abstract] OR "interstitial pneumonias"[Title/Abstract] OR "pneumonias interstitial"[Title/Abstract] OR "pneumonitis interstitial"[Title/Abstract] OR "interstitial pneumonitides"[Title/Abstract] OR "interstitial pneumonitis"[Title/Abstract] OR (("Pneumonia"[MeSH Terms] OR "Pneumonia"[All Fields] OR "Pneumonitides"[All Fields]) AND "Interstitial"[Title/Abstract])) | 108,799 |
| 5 | Diffuse Parenchymal Lung Diseases [Title/Abstract] OR Interstitial Lung Diseases [Title/Abstract] OR Interstitial Lung Disease [Title/Abstract] OR Lung Disease, Interstitial [Title/Abstract] OR Diffuse Parenchymal Lung Disease [Title/Abstract] OR Pneumonia, Interstitial [Title/Abstract] OR Interstitial Pneumonia [Title/Abstract] OR Interstitial Pneumonias [Title/Abstract] OR Pneumonias, Interstitial [Title/Abstract] OR Pneumonitis, Interstitial [Title/Abstract] OR Interstitial Pneumonitides [Title/Abstract] OR Interstitial Pneumonitis [Title/Abstract] OR Pneumonitides, Interstitial [Title/Abstract] | "diffuse parenchymal lung diseases"[Title/Abstract] OR "interstitial lung diseases"[Title/Abstract] OR "interstitial lung disease"[Title/Abstract] OR "lung disease interstitial"[Title/Abstract] OR "diffuse parenchymal lung disease"[Title/Abstract] OR "pneumonia interstitial"[Title/Abstract] OR "interstitial pneumonia"[Title/Abstract] OR "interstitial pneumonias"[Title/Abstract] OR "pneumonias interstitial"[Title/Abstract] OR "pneumonitis interstitial"[Title/Abstract] OR "interstitial pneumonitides"[Title/Abstract] OR "interstitial pneumonitis"[Title/Abstract] OR (("Pneumonia"[MeSH Terms] OR "Pneumonia"[All Fields] OR "Pneumonitides"[All Fields]) AND "Interstitial"[Title/Abstract]) | 35,263 |
| 4 | Interstitial Lung Diseases[MeSH Terms] | "lung diseases, interstitial"[MeSH Terms] | 92,296 |
| 3 | (Dermatomyositis[MeSH Terms]) OR (Dermatomyositis [Title/Abstract] OR Polymyositis-Dermatomyositis [Title/Abstract] OR Polymyositis Dermatomyositis [Title/Abstract] OR Dermatopolymyositis [Title/Abstract] OR Dermatomyositis, Adult Type [Title/Abstract] OR Adult Type Dermatomyositis [Title/Abstract] OR Dermatomyositis, Childhood Type [Title/Abstract] OR Childhood Type Dermatomyositis [Title/Abstract] OR Juvenile Dermatomyositis [Title/Abstract] OR Dermatomyositis, Juvenile [Title/Abstract] OR Juvenile Myositis [Title/Abstract] OR Myositis, Juvenile [Title/Abstract]) | "Dermatomyositis"[MeSH Terms] OR ("Dermatomyositis"[Title/Abstract] OR "Polymyositis-Dermatomyositis"[Title/Abstract] OR "Polymyositis-Dermatomyositis"[Title/Abstract] OR "Dermatopolymyositis"[Title/Abstract] OR (("Dermatomyositis"[MeSH Terms] OR "Dermatomyositis"[All Fields]) AND "adult type"[Title/Abstract]) OR "adult type dermatomyositis"[Title/Abstract] OR "dermatomyositis childhood type"[Title/Abstract] OR "childhood type dermatomyositis"[Title/Abstract] OR "juvenile dermatomyositis"[Title/Abstract] OR "dermatomyositis juvenile"[Title/Abstract] OR "juvenile myositis"[Title/Abstract] OR "myositis juvenile"[Title/Abstract]) | 14,357 |
| 2 | Dermatomyositis [Title/Abstract] OR Polymyositis-Dermatomyositis [Title/Abstract] OR Polymyositis Dermatomyositis [Title/Abstract] OR Dermatopolymyositis [Title/Abstract] OR Dermatomyositis, Adult Type [Title/Abstract] OR Adult Type Dermatomyositis [Title/Abstract] OR Dermatomyositis, Childhood Type [Title/Abstract] OR Childhood Type Dermatomyositis [Title/Abstract] OR Juvenile Dermatomyositis [Title/Abstract] OR Dermatomyositis, Juvenile [Title/Abstract] OR Juvenile Myositis [Title/Abstract] OR Myositis, Juvenile [Title/Abstract] | "Dermatomyositis"[Title/Abstract] OR "Polymyositis-Dermatomyositis"[Title/Abstract] OR "Polymyositis-Dermatomyositis"[Title/Abstract] OR "Dermatopolymyositis"[Title/Abstract] OR (("Dermatomyositis"[MeSH Terms] OR "Dermatomyositis"[All Fields]) AND "adult type"[Title/Abstract]) OR "adult type dermatomyositis"[Title/Abstract] OR "dermatomyositis childhood type"[Title/Abstract] OR "childhood type dermatomyositis"[Title/Abstract] OR "juvenile dermatomyositis"[Title/Abstract] OR "dermatomyositis juvenile"[Title/Abstract] OR "juvenile myositis"[Title/Abstract] OR "myositis juvenile"[Title/Abstract] | 12,724 |
| 1 | Dermatomyositis[MeSH Terms] | "dermatomyositis"[MeSH Terms] | 9,776 |

# Supplementary Table 2: Basic Characteristics of Included Studies

| **Author, year** | **Study design** | **Country** | **Observation**  **period** | **Age (years)** | **Sample**  **size** | **Outcome** | **Diagnostic criteria for RP-ILD** | **Incidence of worsening** | **Influencing factors** |
| --- | --- | --- | --- | --- | --- | --- | --- | --- | --- |
| Wu Y, 2025 | Retrospective | China | 2019.3-2024.3 | 55.4±2.6 | 71 | RP-ILD | RP-ILD was defined as meeting any of the following within 1 month of disease onset: ① acute progressive dyspnea requiring hospitalization or oxygen therapy; ② FVC reduction>10% or DLCO decrease>15% (with reduced FVC); ③ HRCT showing>20% increased extent of interstitial lung abnormalities; ④ arterial blood gas abnormalities (respiratory failure or PaO₂ decrease >10 mmHg) | 30.0%（21/71） | Anti-MDA5 lgG1 IIF, Anti-MDA5 lgG3 IIF, Anti-MDA5 lgG IB, Anti-Ro52 lgG IB, Age≥50 years, Gender(male), NLR^high25.22^, CRP>8 mg/L |
| Wang L, 2024 | Retrospective | China | 2019.3-2021.3 | - | 255 | RP-ILD | RP-ILD was defined as meeting any of the following within 1 month of disease onset: ① acute progressive dyspnea requiring hospitalization or oxygen therapy; ② FVC reduction>10% or DLCO decrease>15% (with reduced FVC); ③ HRCT showing>20% increased extent of interstitial lung abnormalities; ④ arterial blood gas abnormalities (respiratory failure or PaO₂ decrease >10 mmHg) | 36.86%（94/255） | Sex (male), Short disease duration (less than 3 months), AST abnormal, LDH abnormal, CK abnormal, CRP abnormal, SF abnormal, Anti-Ro52 antibody positive, Anti-MDA5 antibody high titer (+++) |
| So J, 2022 | Retrospective | China | 2015.1-2020.12 | 52.0±13.0 | 116 | RP-ILD | Progressive radiological interstitial changes, progressive dyspnea, and hypoxemia within 1 month after the onset of respiratory symptoms (regardless of treatment status) | 40.5%（47/116） | Age at diagnosis>50 years, Sex, Fever, Infection at diagnosis, V sign, NLR>7.0, LDH>300 IU/L at diagnosis, CRP>18mg/dL at diagnosis |
| Shi Y, 2024 | Retrospective | China | - | 53.94±13.46 | 39 | RP-ILD | RP-ILD was defined as meeting any of the following within 1 month of disease onset: ① acute progressive dyspnea requiring hospitalization or oxygen therapy; ② FVC reduction>10% or DLCO decrease>15% (with reduced FVC); ③ HRCT showing>20% increased extent of interstitial lung abnormalities; ④ arterial blood gas abnormalities (respiratory failure or PaO₂ decrease >10 mmHg | 51.28%（20/39） | CRP>8 mg/L, BAFF>2971.5 pg/ml |
| Niu Y, 2024 | Retrospective | China | 2017.1-2022.12 | 53.6±10.4 | 168 | RP-ILD | Acute exacerbation of dyspnea, significant decline in pulmonary ventilation/diffusion function, progressive HRCT findings, and acute respiratory failure within 1 month. | 72.0%（121/168） | IL-6 (pg/mL), Lymphocytes (×109/L) |
| Lv C, 2023 | Retrospective | China | 2019.3-2021.2 | 53.1±12.4 | 246 | RP-ILD | Progressive dyspnea, progressive hypoxemia, and worsening interstitial changes on chest CT within 1 month after the onset of respiratory symptoms | 35.8%（88/246） | CRP≥8mg/L, Disease duration≤3 mo |
| Li M, 2023 | Retrospective | China | 2017.3-2021.12 | 50.5±11.5 | 73 | RP-ILD | Deterioration of imaging findings/pulmonary symptoms, or significant decline in lung function (FVC reduction>10%, PaO₂ decrease>10 mmHg) within 3 months. | 29.58%（21/73） | WBC(×10^9^/L), Lymphocyte (×10^9^/L), LDH>356.15U/L, AST>71.27U/L, ALT>27.75U/L, Albumin>30.9g/L, PNI>34.10 |
| Zou R, 2023 | Retrospective | China | 2017.1-2021.12 | 53.98±10.56 | 105 | RP-ILD | Meeting any of the following within 3 months of respiratory symptom onset: ① acute progressive dyspnea requiring hospitalization/oxygen therapy; ② FVC reduction>10% or DLCO decrease>15%; ③ >20% increased extent of interstitial lesions on HRCT; ④ respiratory failure or PaO₂ decrease>10 mmHg. | 62.8%（66/105） | Age> 53years, Gender, Typical rash,Fever, Arthralgia/arthritis, Lymphocyte count≤ 740 cells/μL, LDH > 307 U/L, Oxygenation index |
| Cheng L, 2025 | Retrospective | China | 2014.12-2022.2 | - | 207 | RP-ILD | RP-ILD was defined as meeting any of the following within 1 month of disease onset: ① acute progressive dyspnea requiring hospitalization or oxygen therapy; ② FVC reduction>10% or DLCO decrease>15% (with reduced FVC); ③ HRCT showing>20% increased extent of interstitial lung abnormalities; ④ arterial blood gas abnormalities (respiratory failure or PaO₂ decrease >10 mmHg | 42.0%（87/207） | Gender (male), Rash, Arthritis, Anti-Ro52 antibody positivity |
| You H, 2023 | Retrospective | China |  | 53.25±12.52 | 272 | RP-ILD | RP-ILD was defined as meeting any of the following within 1 month of disease onset: ① acute progressive dyspnea requiring hospitalization or oxygen therapy; ② FVC reduction>10% or DLCO decrease>15% (with reduced FVC); ③ HRCT showing>20% increased extent of interstitial lung abnormalities; ④ arterial blood gas abnormalities (respiratory failure or PaO₂ decrease >10 mmHg | 33.82%（92/272） | Disease duration＜3months, CRP(>8mg/L), Anti-Ro52 positive, Anti-MDA5 titer++, Anti-MDA5 titer+++ |
| Yan W, 2024 | Retrospective | China | - | 55.78±11.03 | 40 | RP-ILD | RP-ILD was defined as meeting any of the following within 1 month of disease onset: ① acute progressive dyspnea requiring hospitalization or oxygen therapy; ② FVC reduction>10% or DLCO decrease>15% (with reduced FVC); ③ HRCT showing>20% increased extent of interstitial lung abnormalities; ④ arterial blood gas abnormalities (respiratory failure or PaO₂ decrease >10 mmHg | 50.0%（20/40） | Male sex(%),Age(year), ALT U/L, AST U/L, S100A6 100pg/mL |
| Guo L, 2023 | Retrospective | China | 2018.12-2021.12 | - | 254 | RP-ILD | Worsening dyspnea with>10% progression of CT lesions within 1 month, or PaO₂ <60 mmHg within 3 months. | 38.0%（97/254） | WDFY4 rs7919656, FVC% predicted<50%, DLCO% predicted<30%, Prednisolone≥67.5(mg/d), Serum ferrtin≥823(ng/ml) |
| Zuo Y, 2022 | Retrospective | China | 2013.7-2019.2 | 49.7±12.3 | 175 | RP-ILD | Meeting≥2 of the following within 3 months: ① worsening dyspnea; ② increased parenchymal lesions on HRCT; ③ vital capacity (VC) reduction>10% or partial pressure of arterial oxygen (PaO₂) decrease>1.33 kPa. | 62.9%（110/175） | Arthralgia, Fever, Elevated ALT, Elevated LDH, Elevated Fet, Lymphopenia, Decreased CD3+ T, Decreased CD3+CD4+ T, Decreased CD3+CD8+ T, Elevated B2, Elevated CEA, Elevated CA153 |
| Han Y, 2023 | Retrospective | China | 2018.1-2021.9 | 49.39±11.23 | 145 | RP-ILD | Progressive worsening of dyspnea accompanied by hypoxemia and significant aggravation of radiological interstitial lung disease within 3 months | 22.07%  （32/145） | Lactate dehydrogenase (≥370 IU/L), Myasthenia (yes), C-reactive protein (≥5 mg/L), Carcinoembryonic antigen (≥5 ng/mL), Neutrophil/lymphocyte ratio (≥4) |
| Zhang C, 2026 | Retrospective | China | 2017.6-2023.3 | 54.0±13.0 | 67 | RP-ILD | Progressive worsening of dyspnea accompanied by hypoxemia (resting PaO₂ < 80 mmHg) and new bilateral ground-glass opacities/consolidation on HRCT within 3 months after the onset of pulmonary symptoms. | 52.24%（35/67） | Gender, Age of onset, Nor LLZ, Con LMZ, Fever, CRP |

# Supplementary Table 3: Results of Newcastle-Ottawa quality assessment Scale for each included study

| **First author, Year** | **Representativeness of the Exposed Cohort** | **Selection of the Non-Exposed Cohort** | **Ascertainment of Exposure** | **Demonstration That Outcome of Interest Was Not Present at Start of Study** | **Comparability of Cohorts on the Basis of the Design or Analysis** | **Assessment of Outcome** | **Was Follow-Up Long Enough for Outcomes to Occur** | **Adequacy of Follow Up of Cohorts** | **Total**  **score** |
| --- | --- | --- | --- | --- | --- | --- | --- | --- | --- |
| Wu Y, 2025 | 1 | 1 | 1 | 1 | 2 | 1 | 1 | 0 | 8 |
| Wang L, 2024 | 1 | 1 | 1 | 1 | 2 | 1 | 1 | 1 | 9 |
| So J, 2022 | 1 | 1 | 1 | 1 | 1 | 1 | 1 | 0 | 7 |
| Shi Y, 2024 | 1 | 1 | 1 | 1 | 1 | 1 | 1 | 0 | 7 |
| Niu Y, 2024 | 1 | 1 | 1 | 1 | 2 | 1 | 1 | 1 | 9 |
| Lv C, 2023 | 1 | 1 | 1 | 1 | 2 | 1 | 1 | 0 | 8 |
| Li M, 2023 | 1 | 1 | 1 | 1 | 1 | 1 | 1 | 0 | 7 |
| Zou R, 2023 | 1 | 1 | 1 | 1 | 2 | 1 | 1 | 0 | 8 |
| Cheng L, 2025 | 1 | 1 | 1 | 1 | 2 | 1 | 1 | 0 | 8 |
| You H, 2023 | 1 | 1 | 1 | 1 | 2 | 1 | 1 | 1 | 9 |
| Yan W, 2024 | 1 | 1 | 1 | 1 | 1 | 1 | 1 | 0 | 7 |
| Guo L, 2023 | 1 | 1 | 1 | 1 | 2 | 1 | 1 | 0 | 8 |
| Zuo Y, 2022 | 1 | 1 | 1 | 1 | 2 | 1 | 1 | 0 | 8 |
| Han Y, 2023 | 1 | 1 | 1 | 1 | 1 | 1 | 1 | 0 | 7 |
| Zhang C, 2026 | 1 | 1 | 1 | 1 | 2 | 1 | 1 | 0 | 8 |

# Meta-Analysis Results

## Demographic characteristics


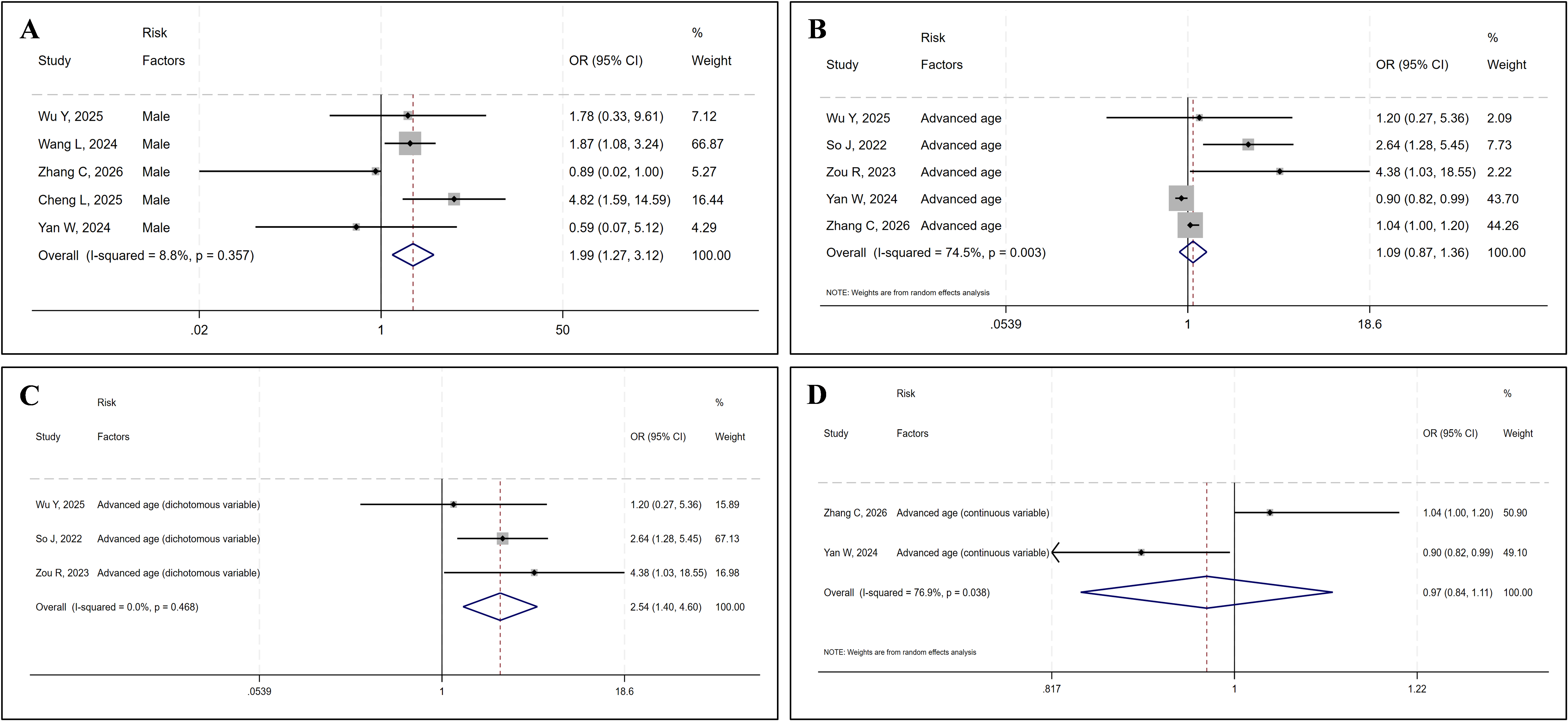


**Figure 1** Forest plot of demographic characteristics (A: Male; B: Advanced age; C: Advanced age [dichotomous variable]; D: Advanced age [continuous variable])

## Clinical characteristics


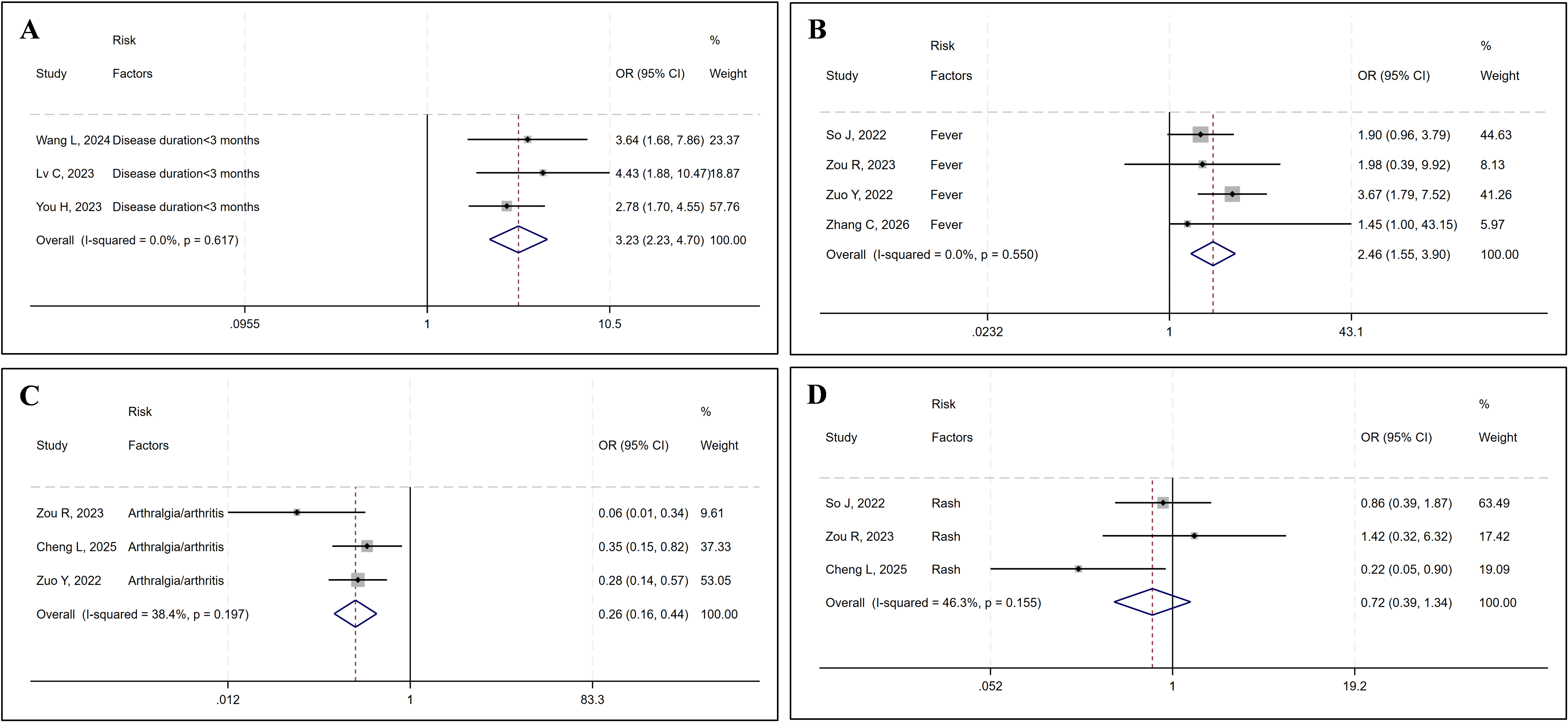


**Figure 2** Forest plot of clinical characteristics (A: Disease duration<3 months; B: Fever; C: Arthralgia /arthritis; D: Rash)

## Immunological marker


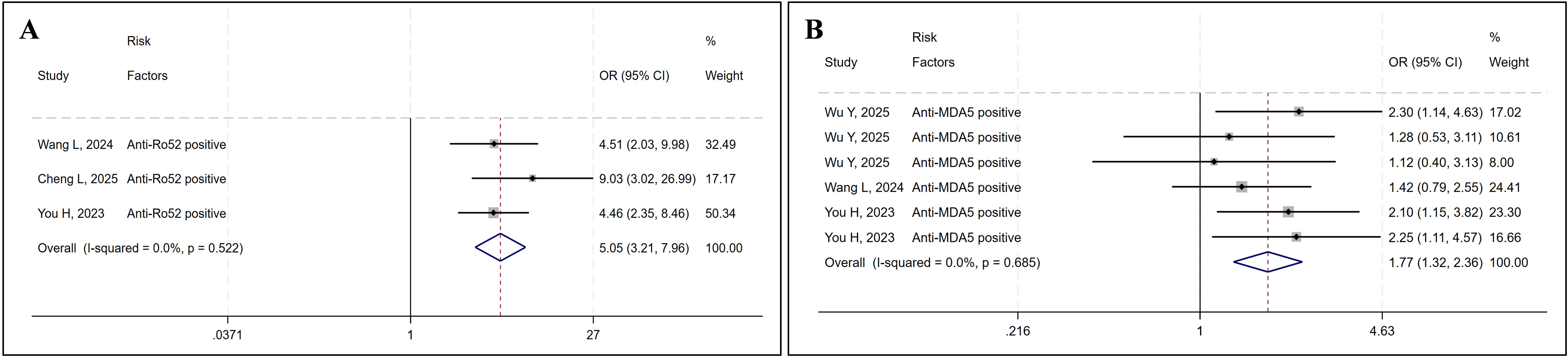


**Figure 3** Forest plot of immunological marker (Anti-Ro52 positive)

## Laboratory data


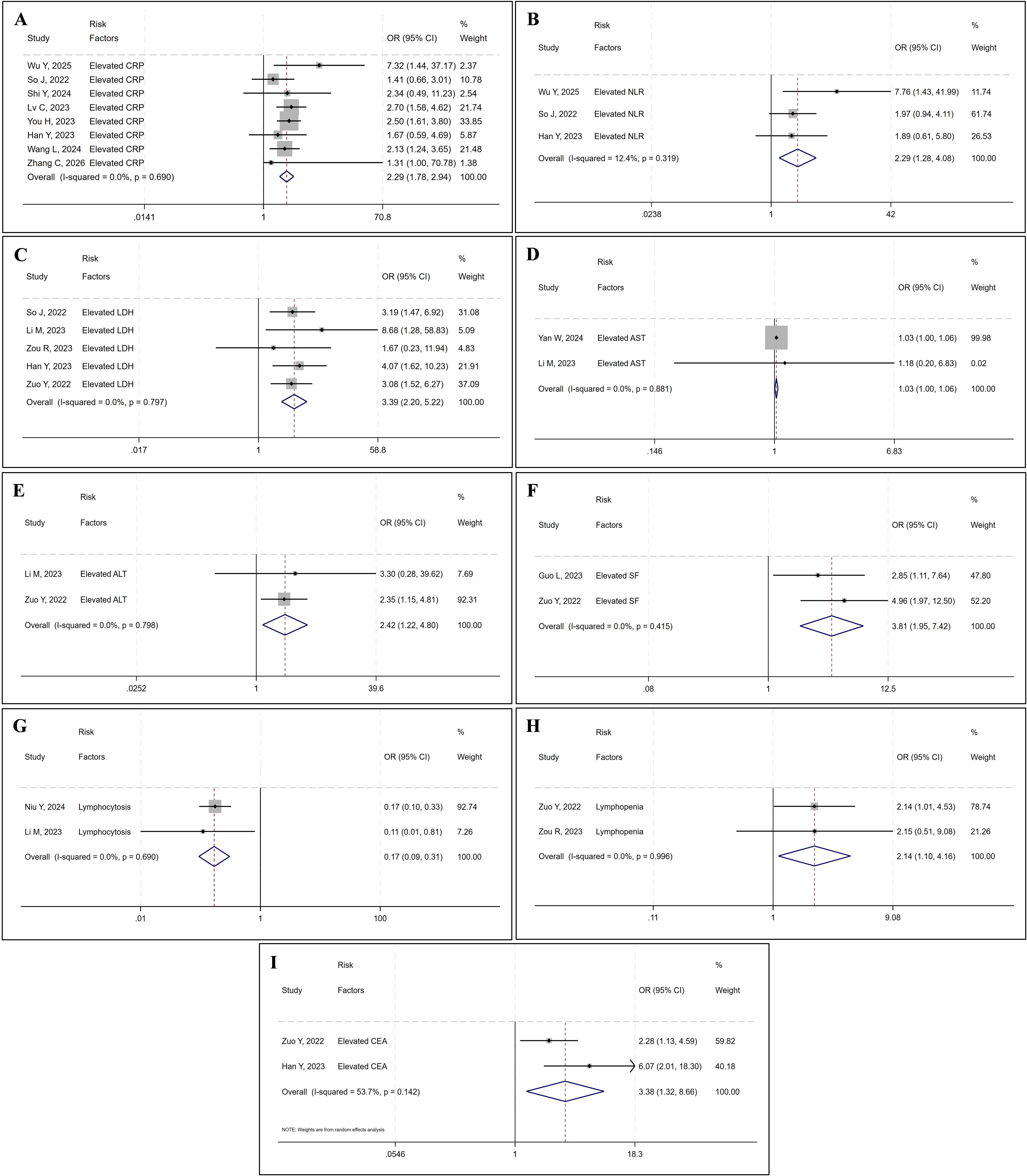


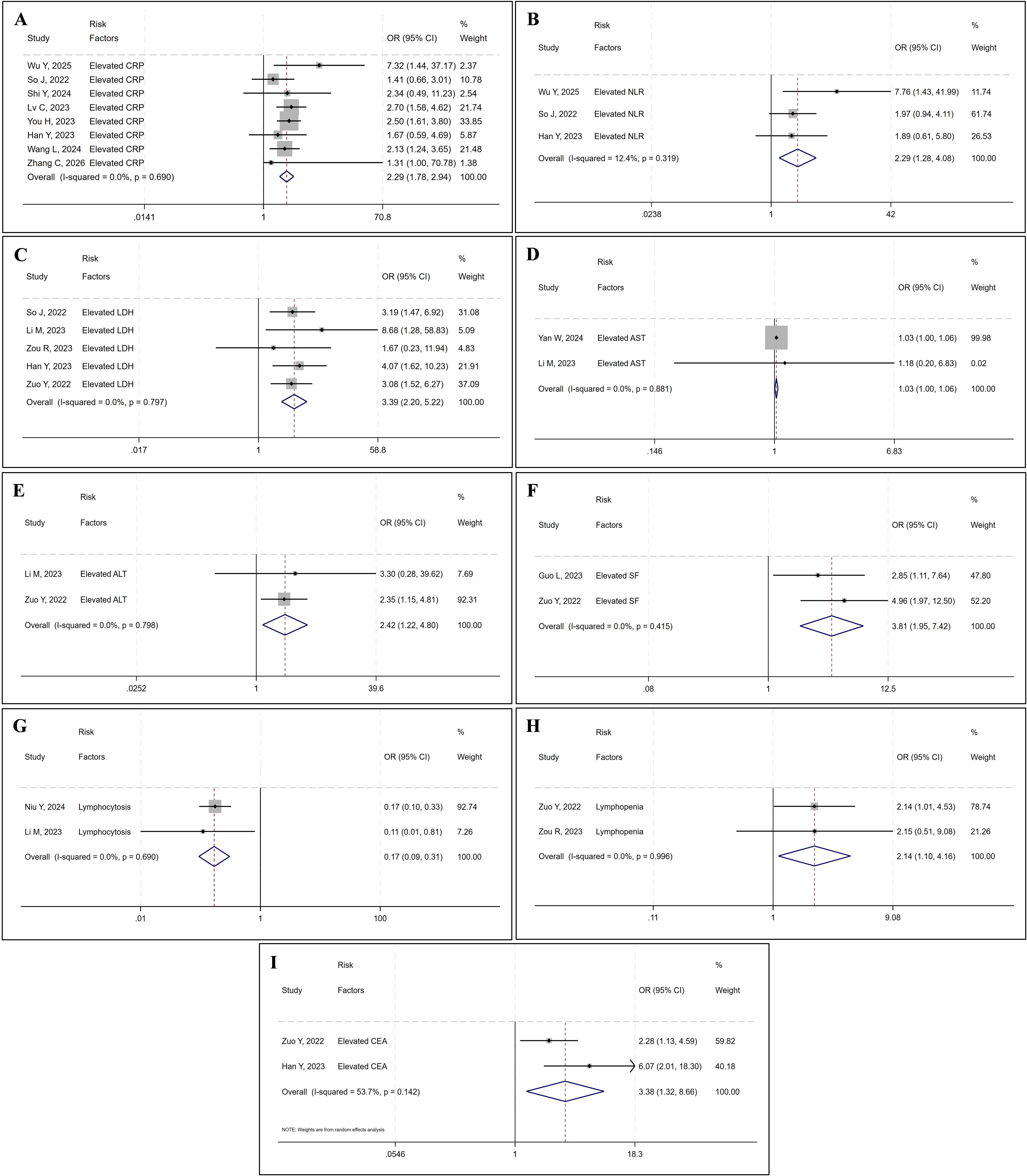


**Figure 4** Forest plot of laboratory data (A: Elevated CRP; B: Elevated NLR; C: Elevated LDH; D: Elevated AST, E: Elevated ALT, F: Elevated SF, G: Lymphocytosis, H: Lymphopenia, I: Elevated CEA)

# Sensitivity analysis

## Demographic characteristics


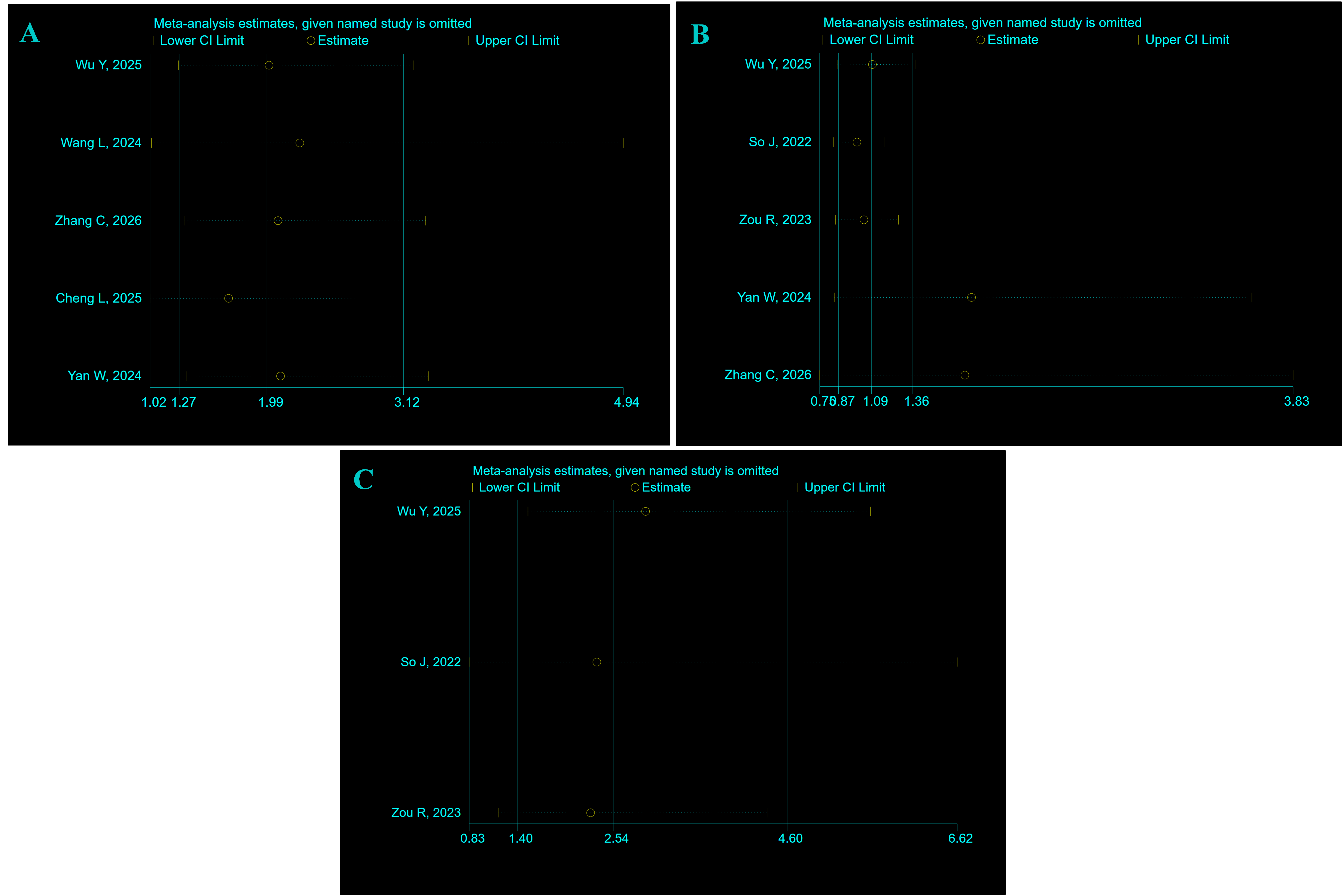


**Figure 5** Sensitivity analysis of demographic characteristics (A: Male; B: Advanced age; C: Advanced age [dichotomous variable])

## Clinical characteristics


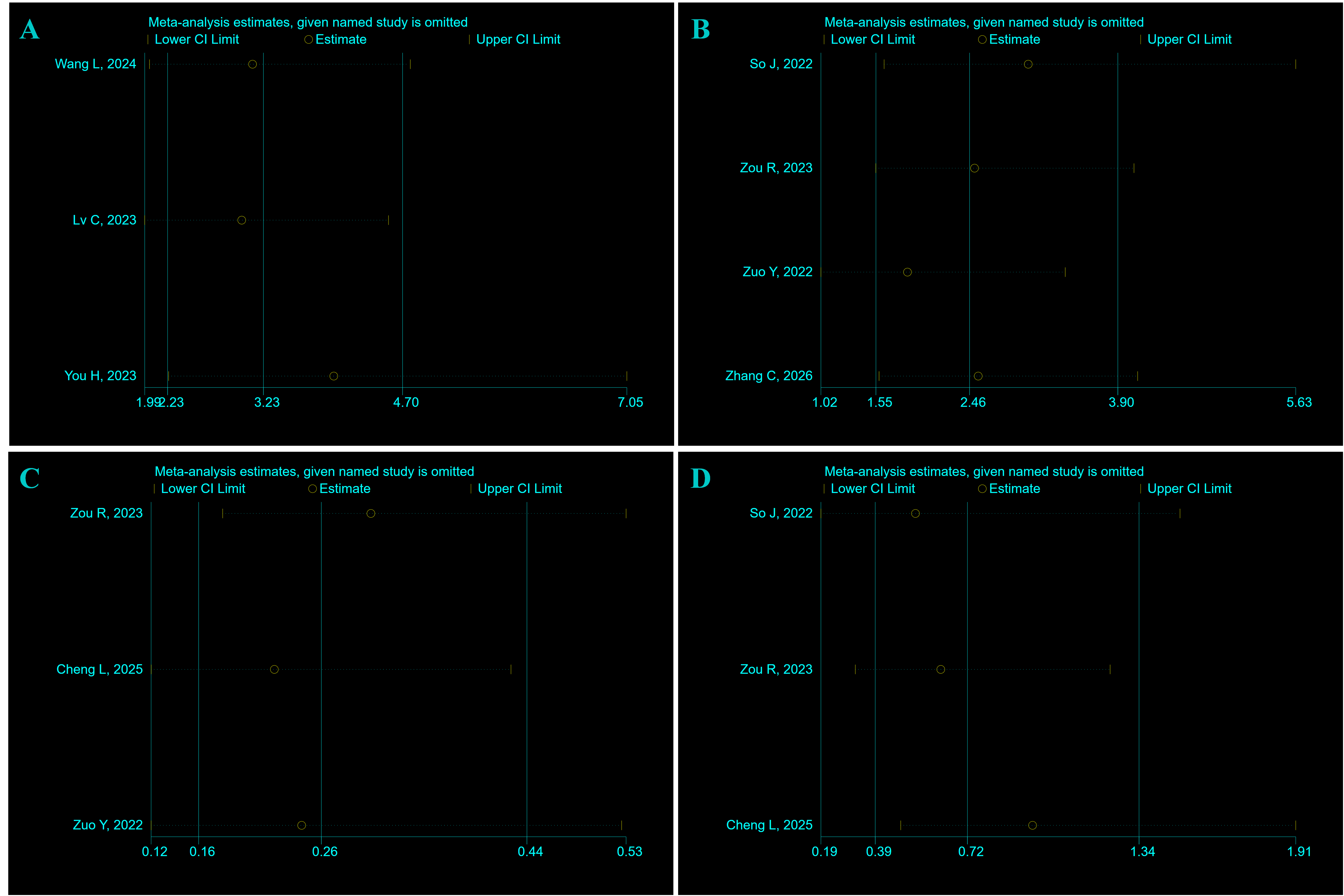


**Figure 6** Sensitivity analysis of clinical characteristics (A: Disease duration<3 months; B: Fever; C: Arthralgia/arthritis; D: Rash)

## Immunological marker


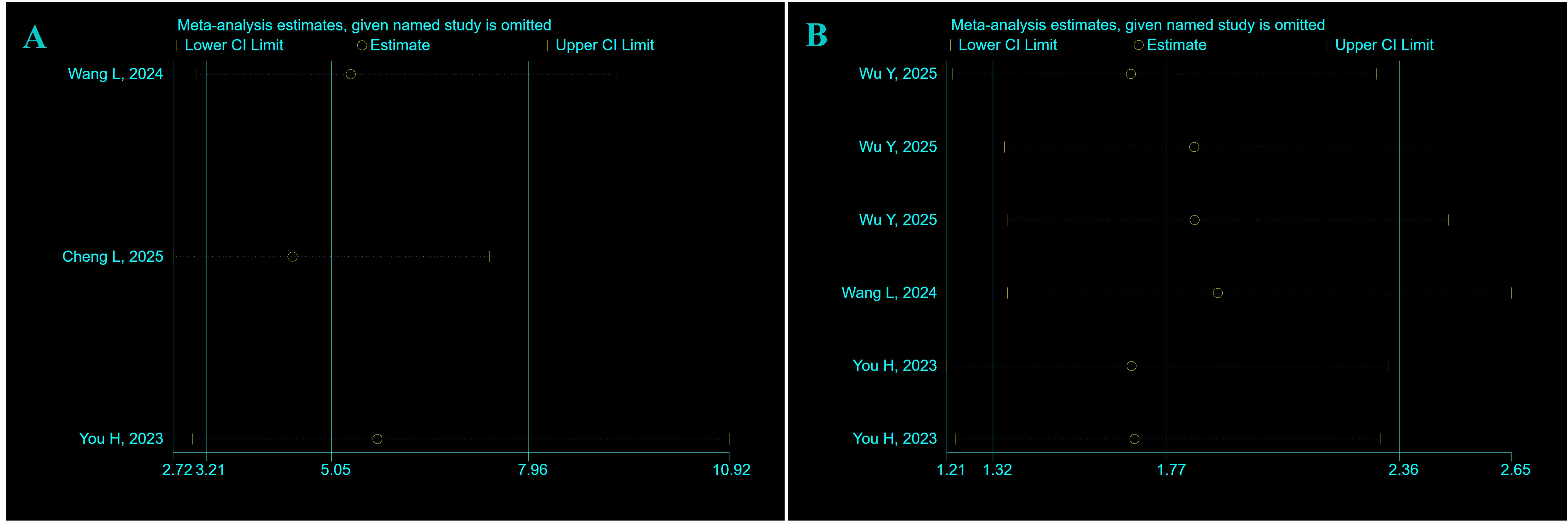


**Figure 7** Sensitivity analysis of immunological marker (A: Anti-Ro52 positive; B: Anti-MDA5 positive)

## Laboratory data


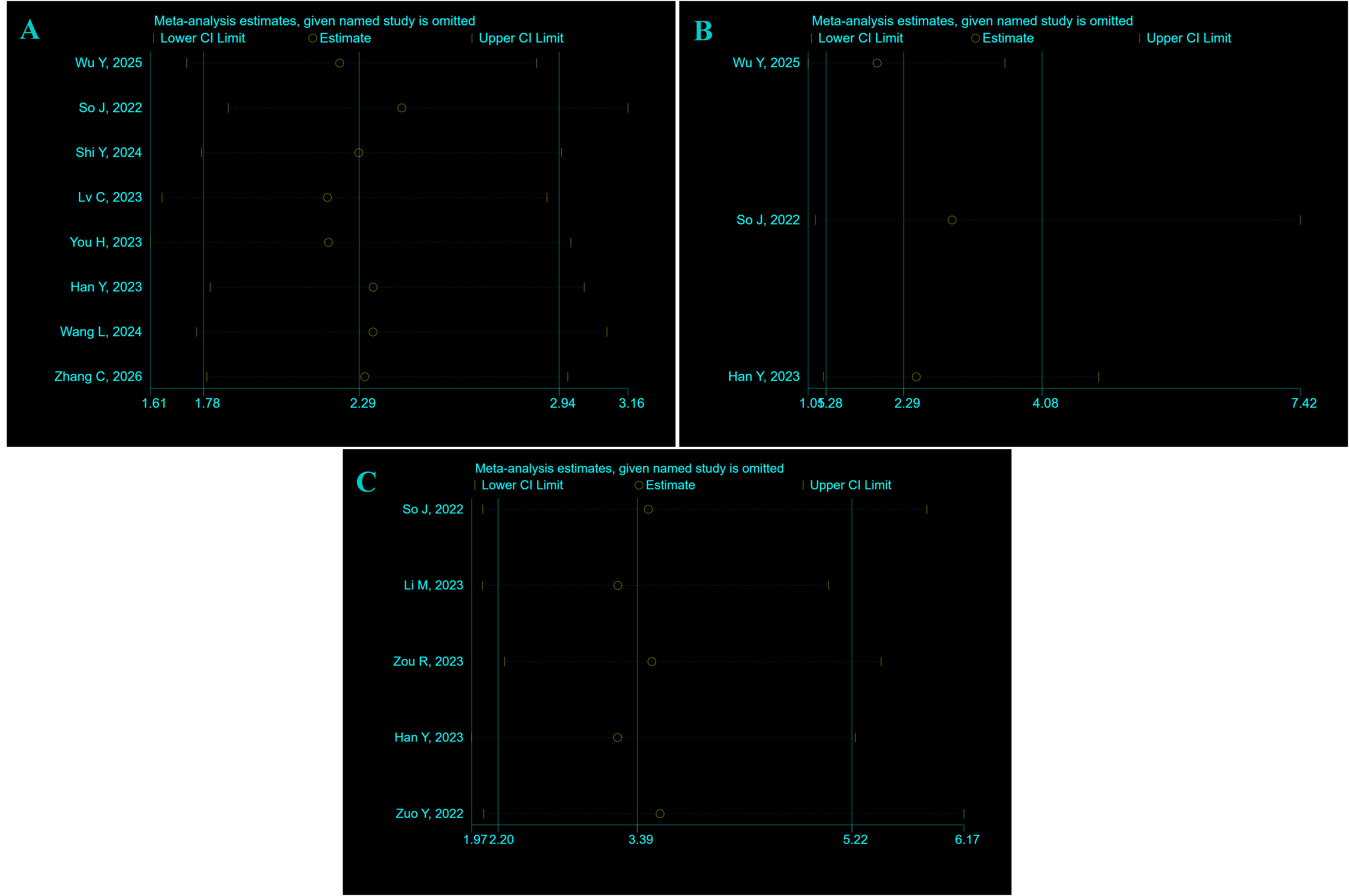


**Figure 8** Sensitivity analysis of laboratory data (A: Elevated CRP; B: Elevated NLR; C: Elevated LDH)

# Bias Assessment

## Demographic characteristics


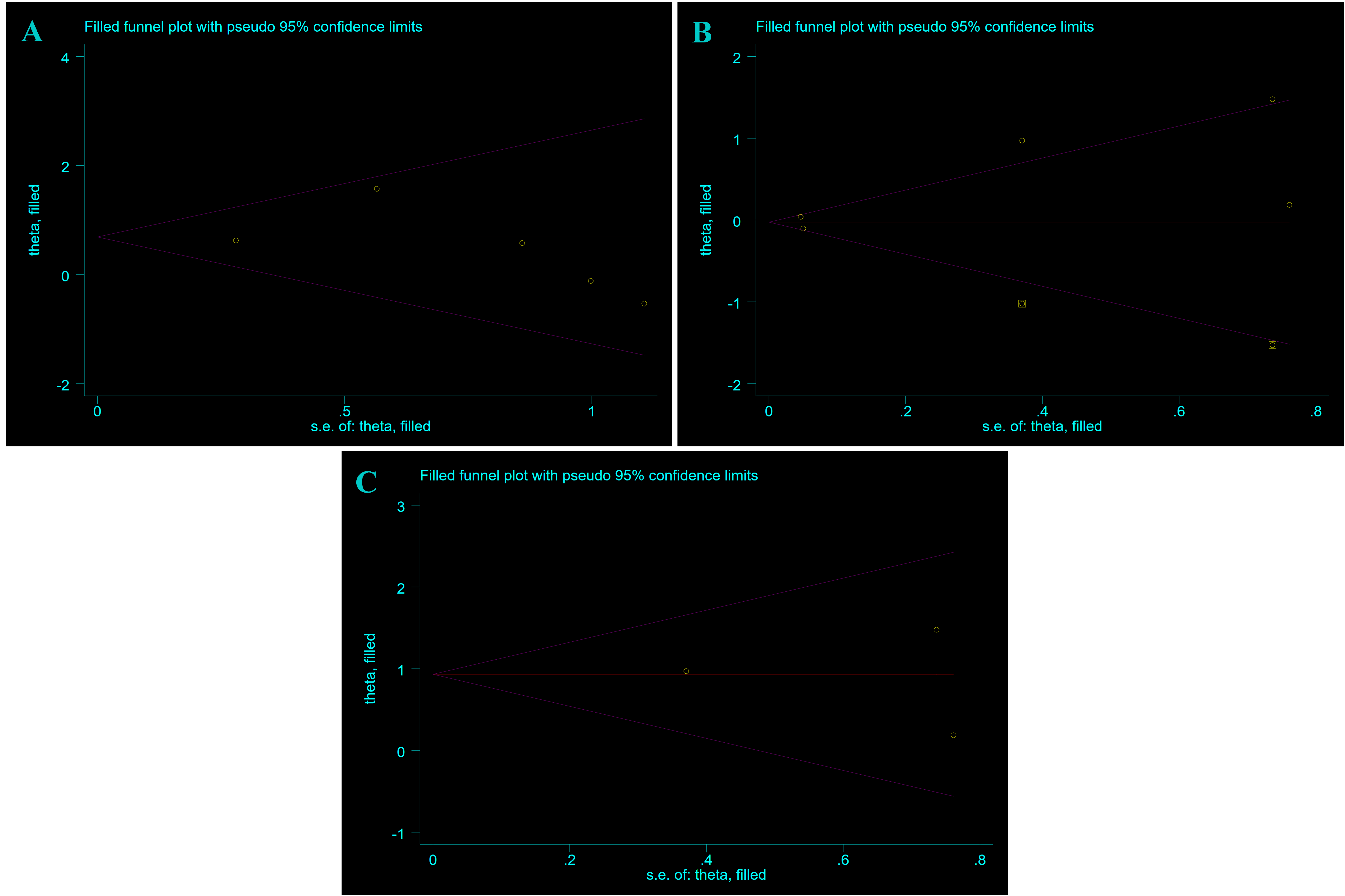


**Figure 9** Funnel plot of demographic characteristics using the trim-and-fill method (A: Male; B: Advanced age; C: Advanced age [dichotomous variable])

## Clinical characteristics


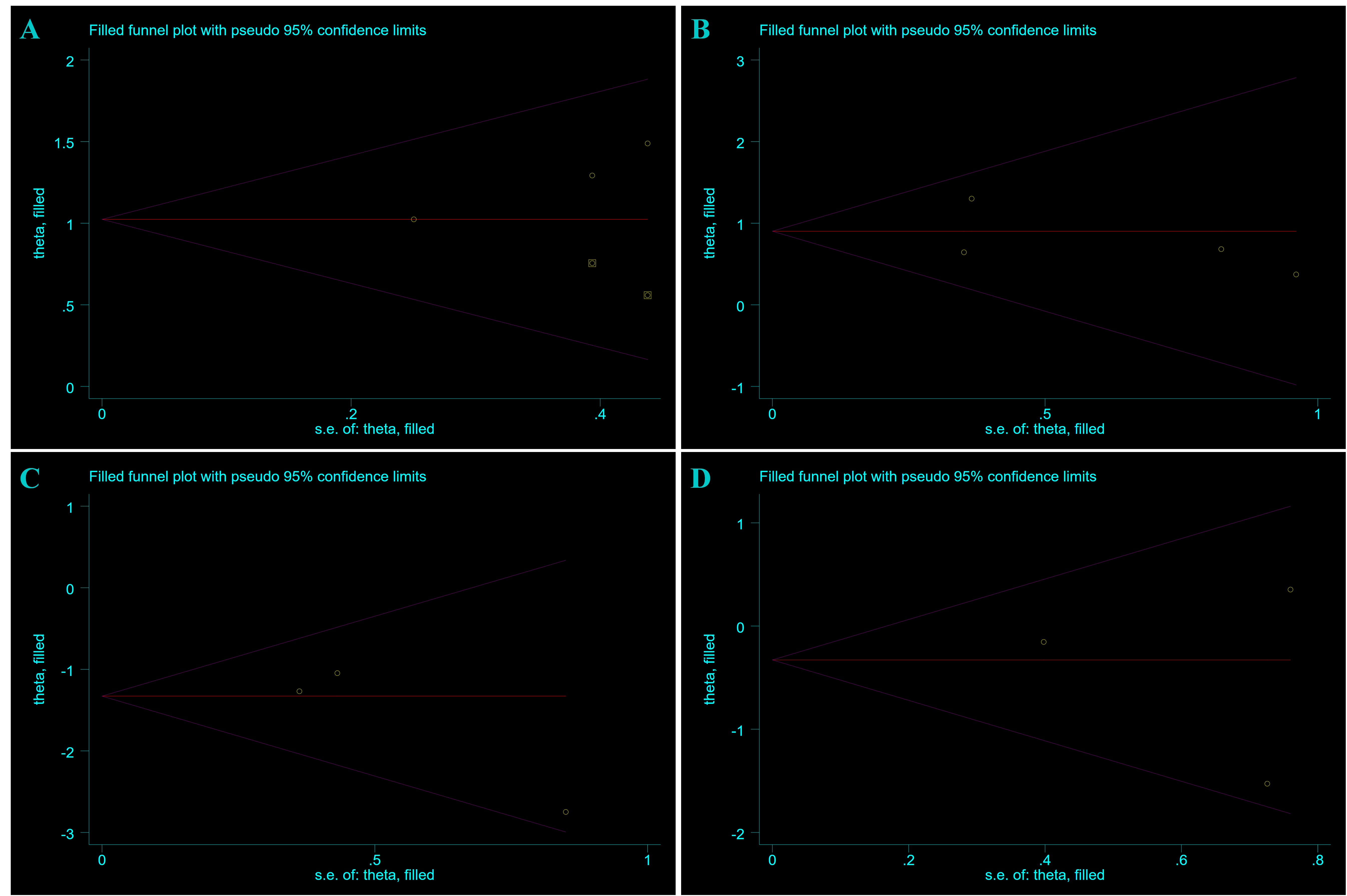


**Figure 10** Funnel plot of clinical characteristics using the trim-and-fill method (A: Disease duration<3 months; B: Fever; C: Arthralgia/arthritis; D: Rash)

## Immunological marker


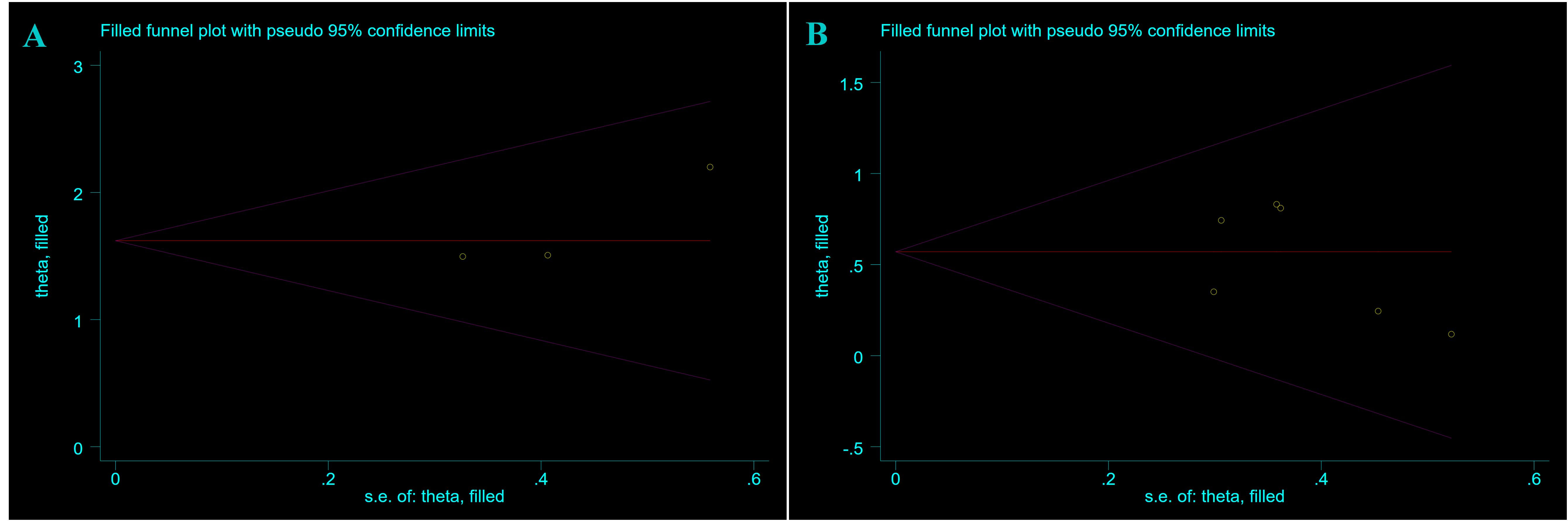


**Figure 11** Funnel plot of immunological marker using the trim-and-fill method (A: Anti-Ro52 positive; B: Anti-MDA5 positive)

## Laboratory data


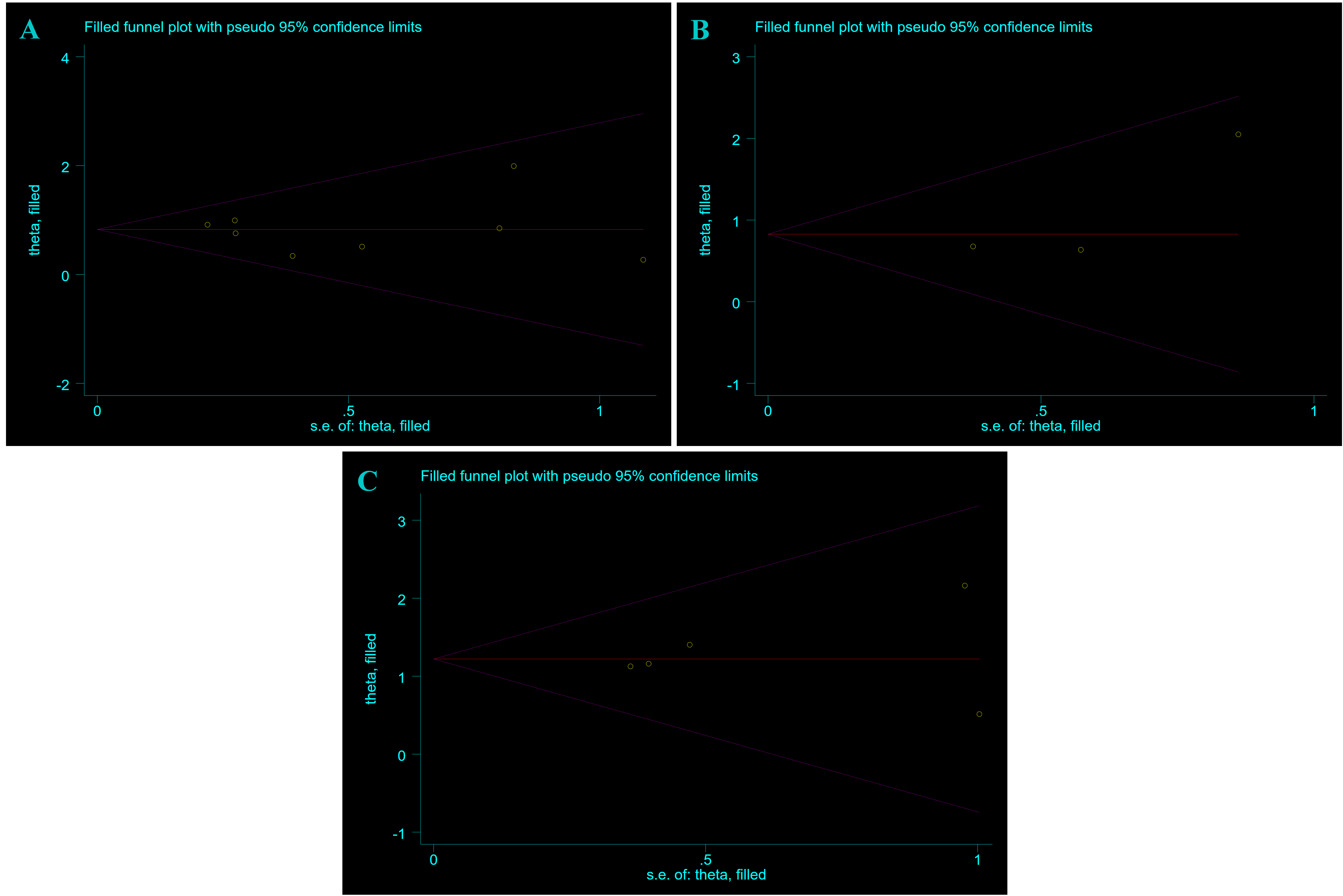


**Figure 12** Funnel plot of laboratory data using the trim-and-fill method (A: Elevated CRP; B: Elevated NLR; C: Elevated LDH)
